# Novel purine derivatives mitigate hypoxia ischemia related brain injury through agrin, zyxin and synaptotagmin proteins

**DOI:** 10.1016/j.neurot.2025.e00621

**Published:** 2025-06-17

**Authors:** Aloïse Mabondzo, Clémence Disdier, Amal Bouzid, Khadidja Side Larbi, Amalia Tsintzou, Auriane Maïza, Boram Kim, Narciso Costa, Rania Harati, Anvi Laetitia Nguyen, Alain Pruvost, Hervé Galons, Nassima Oumata, Jean Armengaud, Marlou Knijnenburg, Gaurav Verma, Henrik Hagberg, Pierre Gressens, Xiaodi F. Chen, Rifat A. Hamoudi, Barbara S. Stonestreet

**Affiliations:** a Université Paris-Saclay, CEA, INRAE, Département Médicaments et Technologies pour la Santé (MTS), SPI, Laboratoire d’Etude de l’Unité Neurovasculaire et Innovation Thérapeutique (LENIT), Gif-sur-Yvette Cedex 91191, France; b Department of Pediatrics, Women & Infants Hospital of Rhode Island, Alpert Medical School of Brown University, Providence, 02905, United States; c Research Institute for Medical and Health Sciences, University of Sharjah, Sharjah, United Arab Emirates; d Department of Pharmacy Practice and Pharmacotherapeutics, College of Pharmacy, University of Sharjah, Sharjah, United Arab Emirates; e Paris Cité University, Faculté des Sciences Pharmaceutiques et Biologiques, 4, Avenue de l’Observatoire, Paris Cedex 06, 75270, France; f Université Paris-Saclay, CEA, INRAE, Département Médicaments et Technologies pour la Santé (DMTS), SPI, Bagnols-sur-Cèze, 30200, France; g Centre of Perinatal Medicine and Health, Institute of Clinical Sciences, University of Gothenburg, Sweden; h Centre of Excellence for Precision Medicine, Research Institute for Health and Medical Sciences, University of Sharjah, Sharjah, United Arab Emirates; i Université Paris Cité, NeuroDiderot, Inserm, F-75019 Paris, France; j BIMAI-Lab, Biomedically Informed Artificial Intelligence Laboratory, University of Sharjah, Sharjah, United Arab Emirates; k Division of Surgery and Interventional Science, University College London, London, United Kingdom

**Keywords:** Blood-brain barrier, Brain injury, Hypoxia-ischemia, Mitochondrial targets, Neuroprotection, Purine derivative drug candidate

## Abstract

Hypoxic-ischemic encephalopathy (HIE) is a major cause of morbidity and mortality in newborns resulting in motor and cognitive impairment. Therapeutic hypothermia is the only treatment approved for HIE. Consequently, there is a critical requirement for additional treatments for hypoxic-ischemic (HI) brain injury because hypothermia is only partially protective. Pharmacological therapeutics are as yet not available to treat HIE. Therefore, we developed a novel trisubstituted purine-derivative drug (BRT_002) to attenuate HI related brain injury. The safety of BRT_002 was confirmed by treating adult rats with BRT_002 (100 ​mg/kg) for 7 days. Postnatal day-7 rats exposed to sham surgery or carotid ligation and 8% FiO_2_ for 90 ​min were given BRT_002 (30 ​mg/kg) or placebo intraperitoneally (IP) immediately, 24, and 48 ​h after the induction of HI. Pharmacokinetic studies revealed suitable systemic and brain exposure to BRT_002. Treatment with BRT_002 reduced neuropathological infarct volumes in the neonatal rats. Bioinformatics analyses of proteomic data identified upregulation of Agrin, Zyxin and Syt5 (p ​< ​0.05) in both brain hemispheres in the male and female neonatal rats after treatment with BRT_002. BRT_002 also augmented mitochondrial respiration and produced metabolic changes in mouse neurons exposed to oxygen-glucose deprivation *in vitro*. Protein-protein interactions suggest that Syt5 interacts with major participants required to attenuate injury and/or facilitate parenchymal brain repair through Fblim1 that include Agrin, Zyxin, Vegfa, Vwf and mitochondrial targets. Our study provides preclinical findings that could serve as a foundation for future clinical trials of this novel purine derivative for the treatment of newborns exposed to HIE.

## Introduction

Hypoxic–ischemic encephalopathy (HIE) is a major cause of neurodevelopmental morbidities in full-term infants [1]. HIE can result from a variety of pregnancy-related disorders before or during birth including uterine rupture, umbilical cord entrapment, placental insufficiency, and pre-eclampsia. HIE occurs in one to three per 1000 live births in high-income countries and 26 per 1000 live births in low-resource countries [2]. The prevalence of HIE in 2019 was estimated to range from 11,000 to 88,000 cases in high-resource countries [[3], [4], [5]]. Brain injury associated with HIE is thought to result from reduced blood flow to the brain combined with lower-than normal concentrations of oxygen in the arterial blood. These events can result in significant mortality and long-term neurological disabilities including cerebral palsy, epilepsy, severe learning and intellectual disabilities, developmental cognitive and motor impairment, behavioral deficits, and later psychiatric disorders [[6], [7], [8], [9]].

Hypothermia is the only therapeutic intervention currently approved for the treatment of HIE in newborn infants. However, this therapeutic modality is only partially protective [10,11], can only be used to treat full-term infants with HIE, and does not improve outcomes after HIE in infants less than 36 weeks of gestation [12]. Infants exposed to moderate or severe HIE often die or have severe residual intellectual disabilities, epilepsy, and/or cerebral palsy even after treatment with therapeutic hypothermia [6,[13], [14], [15]]. Recent findings show that death rates of infants exposed to HIE after treatment with therapeutic hypothermia still range from approximately 9 to 20%, death combined with neurodevelopmental impairment range from 40 to 50%, and residual neurological sequelae range from 20 to 50% in these infants [16]. Moreover, the long-term beneficial effects of hypothermia in these infants remain somewhat controversial [11]. Pharmacological agents are not available as yet to treat HIE despite numerous studies examining the pathophysiology of neonatal brain injury. Although numerous studies investigated potential pharmacological agents to treat HIE in preclinical trials, most of these agents have not been proven to be efficacious in clinical trials [[17], [18], [19], [20]]. Therefore, it is essential to develop alternative and/or adjunctive novel therapeutic agents to attenuate the sequela of HIE. Some previous approaches have not proven successful in part potentially because they have focused upon discrete aspects of the neuropathology originating from hypoxic-ischemic (HI) brain injury such as the neuronal compartment rather than examining potential dynamic interactions within the brain among multiple biological systems.

Given the above considerations, we sought to develop a novel pharmacological agent based upon an innovative trisubstituted purine derivative, which potentially could target multiple biological systems and compartments within the central nervous system (CNS). These novel therapeutic agents have the capacity to penetrate the brain parenchyma and target multiple complex processes that are thought to have critical functions during the evolution of HI-related brain injury including the modulation of inflammation, mitochondrial function, and neuronal injury [[21], [22], [23]]. Purine analogs are well-known as pharmacodynamic entities with the ability to regulate cardiac blood flow and myocardial oxygen consumption [24]. Extensive evidence suggests that reductions in cortical blood flow are associated with reductions in oxygenation during HI [25], which could also target the CNS endothelial cells. Importantly, findings also suggest that CNS endothelial cell dysfunction could impair neuronal activity [26,27].

The primary goal of the current study is to determine whether our newly developed novel purine derivative (BRT_002) agent could attenuate neuropathological brain injury in postnatal day-7 (P7) rats after exposure to HI-related brain injury. The safety and efficacy of BRT_002 were first examined by determining the potential toxicity and pharmacokinetic properties of BRT_002. Proteomics comparisons also were performed between different subgroups of ipsilateral and contralateral brain hemispheres in the BRT_002-treated male and female neonatal rats after exposure to HI. Variable bioinformatic analyses followed by molecular validation were carried out to elucidate potential molecular mechanism(s) fundamental to the potential neuroprotection provided by BRT_002.

## Materials and Methods

### Ethical approval and animal housing

The experimental procedures for pharmacokinetic (PK) studies study were approved by the French Ministry of Health (Authorization number: APAFIS#30306-2021031009268181 v1). The toxicology study was performed according to the prerequisite Good Laboratory Practice requirements. This study was conducted in accordance with Directive 2010/63/UE of the European Parliament and the Council of September 22, 2010, for the protection of animals used for scientific purposes (Approval for the site of experimentation: No. E 18-023-01).

The animal protocols for HI-related brain injury studies were approved by the Institutional Animal Care and Use Committees of the Alpert Medical School of Brown University and Women & Infants Hospital of Rhode Island (Approval number: IACUC# 20-05-0006). Pregnant wild type *Wistar Rattus norvegicus* were obtained from Charles River Laboratories (Wilmington, MA, USA) on embryonic day 15 or 16 and housed in a temperature-controlled facility in the Care Facility at Brown University on a 12-h light/dark cycle with *ad libitum* access to food and water. The dates upon which the rat pups were delivered were confirmed and designated as postnatal (P) day zero. The pups were housed as litters with the dams after birth up to the end of the study. Litters were culled to a maximum of 10 pups on P1 with equal numbers of male and female pups to the extent possible. The pups within each litter were randomly assigned to one of three groups on P7: Vehicle-treated treated sham-operated control (Sham), HI ​+ ​Vehicle-treated, and HI ​+ ​BRT_002-treated groups. The sex of each animal was recorded.

### Design and synthesis of BRT_002

A purine BRT_001 was first developed. However, its low solubility limited its use *in vivo*. It was converted to its valyl ester BRT_002 (Supplementary Fig. 1), which could be used in the animal studies.

### Toxicity examination

The toxicity examination consisted of two stages: an increasing dose phase (stage 1) and a 7-day fixed-dose phase (stage 2). Four groups of three male rats per group were each treated with four increasing doses of BRT_002 in the stage 1 study. Each dose was followed by at least a one-day observation period, after which the subsequent dose was administered. Treatment with BRT_002 was terminated if signs of intolerance occurred or a dose of 100 ​mg/kg was achieved. The animals used in the stage 1 study were observed for an additional 2–3 days before sacrifice after BRT_002 had been administered. Functional and neurobehavioral tests were performed on the day of each treatment for the stage 1 studies, before the first dose and on the last daily dose (day 7) for the phase 2 studies, the animals were observed according to a standardized observation battery for neurobehavioral, neurovegetative, psychotropic signs or neurotoxic effects, and body temperature regulation. The methodology is based upon the Irwin screen modified by suppressing the graduation of intensity of clinical signs. The animals were observed individually in a cage without sawdust in a quiet room. Clinical signs were observed according to the following criteria: behavior (awareness, mood, motor activity, and motor coordination), neurologic profile (muscle tone, body posture, autonomic profile, central excitation, and reflexes). The observation time was approximately 2 ​h after a dose or at the peak of occurrence of any clinical signs if different.

Blood samples for toxicokinetic analysis were obtained from the retro-orbital sinus from the animals after anesthesia with isoflurane inhalation. Sample volumes and anticoagulants were as follows: 0.3 ​mL into EDTA tubes (hematology), 1 ​mL into lithium heparin tubes (for analysis blood chemistry) and 1.3 ​mL into citrated tubes (for coagulation analysis). Hematology, blood chemistry and coagulation analyses were performed as determined by standard general laboratory methodology.

Blood samples (0.3 ​mL) for drug analysis were obtained from animals via the jugular vein or other vessels under isoflurane anesthesia and placed in tubes with lithium heparin. Blood samples were obtained from the treated animals as follows: (i) on day 1: 1, 3, 8 and 24 ​h and (ii) on day 7: before the first dose, and at 1, 2, 3, 4 and 8 ​h after the first dose. Blood samples were taken from the control animals at the following times: (i) on day 1: 1 and 3 ​h and (ii) day 7: before the first dose, and 3 and 8 ​h after the first dose. The blood samples were placed on ice, and plasma obtained within 60 ​min of sampling by centrifugation under refrigeration (+4 ​± ​2 ​°C) for 10 ​min at 1500 ​g. The plasma was placed into polypropylene tubes with 100 ​μL in each tube and frozen within 90 ​min after blood sampling and remained frozen (≤-18 ​°C) for drug analysis.

### HI-related brain injury in neonatal rats

The Vannucci model was used to induce HI-related brain injury [28]. Briefly, rats were anesthetized using 4 ​% isoflurane and maintained with 2 ​% isoflurane. The absence of a leg withdrawal reflex was verified before proceeding with surgery. A midline ventral incision was made on the neck. The right common carotid artery (RCCA) was located and double ligated with a 5-0 silk suture. Sham animals were exposed to the same procedure except the RCCA was not ligated. The neck incision was closed with a 5-0 silk suture. Body temperature was maintained at 37 ​°C during surgery using an isothermal heating pad [29]. The pups were returned to their dams for 1.5–3 ​h of recovery, then the animals in the HI-treated groups were placed in a hypoxia chamber (Biospherix, Parish, NY, USA) with 8 ​% humidified oxygen and balanced with nitrogen for 90 ​min at a rectal temperature of 36 ​°C. Sham animals were exposed to room air for 90 ​min at a temperature of 36 ​°C. The pups were given intraperitoneal (IP) injections of 30 ​mg/kg BRT_002 or an equivalent volume of placebo (normal saline/PEG200) immediately after exposure to HI. Then, the pups were returned to the dams. The HI ​+ ​BRT_002-treated groups were given additional 30 ​mg/kg IP doses of BRT_002 ​at 24, and 48 ​h after termination of HI, and the HI placebo-treated group received vehicle (normal saline/PEG200). The subjects were weighed before surgery and at the time of each HI ​+ ​BRT_002 or placebo injection.

### Behavior analyses

A walking test was conducted on P8, P9 and P10 to determine early locomotor activity in the neonatal rats after exposure to HI-related brain injury with and without treatment with BRT_002. Rats were placed on a surface and observed for 2–3 ​min, and three states of ambulation were determined: crawling, transition or beginning to walk. The animals were crawling when the limbs and tail touched the surface. The pups were considered to be in transition when movement of the back limbs and tail that were slightly elevated above the surface. The rats were beginning to walk when the back limbs and tail are totally elevated above the surface.

The righting reflex test was conducted on P10. The pups were placed in a supine position to determine the ability of the animal to right to a prone position. The animals were retained for 2 ​s in the supine position and then released. The ability to turn over onto all four legs was examined by measuring the time to turn to the prone position. The total testing time was 30 ​s.

The negative geotaxis test was used to examine motor coordination in young rats on P8, P9 and P10. Rats are placed facing downward on a slope, because of gravitational vestibular cues, the pups were expected to turn up the slope. The response to the stimulus is an innate behavior. The pups were placed with the head pointing downward on a 45° incline and held for 5 ​s. They were then released and the time measured for the pups to turn face upward was recorded. The total testing time was 30 ​s. All behavioral studies were performed by investigators who were not aware of the group assignments.

### PK studies in neonatal rats

Neonatal pups were sacrificed at 2, 4, 6 and 24 ​h after treatment with BRT_002 for the PK studies. Blood was collected by cardiac puncture, cerebral spinal fluid (CSF) was obtained with a micropipette, and the brain was perfused with PBS by cardiac puncture. Thereafter, the brains were removed, weighed and frozen. Samples were stored at −80 ​°C until analyses.

Brain samples were homogenized with two volumes of water using a Precellys homogenizer (Bertin Technologies, Montigny-le-Bretonneux, France) to quantify the drug concentration. Five microliters of the internal standard (a compound with a structure similar to that of BRT_002) solution were added to 50 ​μL of rat plasma or brain homogenates, and then 200 ​μL of acetonitrile were added for protein precipitation. The sample was vortexed for 15 ​s and centrifuged for 10 ​min at 20,000 ​g and 5 ​°C. The supernatant was diluted in a one-fifth volume of water and transferred into a new vial for analysis. Five microliters of extract maintained at +5 ​°C was injected into an LC‒MS/MS system consisting of a Waters ACQUITY UPLC® System (Waters, Saint-Quentin-en-Yvelines, France) with an Acquity UPLC BEH C18 2.1∗50 ​mm column and run on a reversed-phase gradient for a 5-min run time. A mixture of mobile phase A (water and 0.1 ​% formic acid) and mobile phase B (acetonitrile and 0.1 ​% formic acid) was used at a flow rate of 0.5 ​mL/min and a column temperature of 50 ​°C. The gradient was increased from 5 ​% B to 100 ​% B between 0.5 and 3 ​min, maintained between 3 and 3.5 ​min, decreased to 5 ​% B at 3.51 ​min and maintained for up to 5.0 ​min for re-equilibration. The mass spectrometry (MS) analysis was performed on a Waters XEVO™ TQ-S mass spectrometer (Waters, Saint-Quentin-en-Yvelines, France) operating in positive ion electrospray MRM mode. The multiple transitions monitored were *m*/*z* 482.3 ​> ​364.9, 383.3 ​> ​261.1 and 395.0 ​> ​273.1 for BRT_002 and BRT_001, respectively. The mean retention times were approximately 2.15 and 2.70 for BRT_002 and BRT_001 under these conditions, respectively. Quantification was performed using linear regression with 1/X weighting and calibration ranges from 1.87 to 3215 ​ng/mL and 0.373–641 ​ng/mL for BRT_002 and BRT_001, respectively. A standard calibration curve and quality control samples prepared with a blank matrix spiked with compounds were used.

### Infarct volume determinations

The subjects were sedated with ketamine (74 ​mg/kg, i.p.) and xylazine (4 ​mg/kg, i.p.) at 72 ​h after HI. The brains were perfused with PBS and 4 ​% paraformaldehyde (PFA) via cardiac puncture at a flow rate of 3 ​mL/min. Thereafter, the brains were removed, weighed, post-fixed with PFA for 24 ​h, and stored in 30 ​% sucrose in phosphate buffer (0.1 ​M) at 4 ​°C before cryosectioning. Each brain was divided into four or five 2-mm coronal sections using a brain slicer matrix (Zivic instruments, Pittsburgh, PA, USA), immersed in optimal cutting temperature embedding medium, and frozen in a metal beaker filled with isopentane surrounded by crushed dry ice. Five cryosections (20 ​μm) were obtained from each 2-mm section and mounted on gelatin-coated slides. One of every fourth cryosection was randomly selected for cresyl violet staining to evaluate HI-related brain injury. Images of the hemispheric and damaged areas were obtained using a Micropublisher 6 CCD camera (Qimaging, Surrey, British Columbia, Canada) and analyzed with ImageJ (NIH) by two experimenters, who were not aware of the group assignments. The respective volume of each measured area was calculated by multiplying the distance between sections. The infarct volume was calculated using the indirect method as a percentage of the ratio of the damaged volume to the total volume of the contralateral hemisphere with correction for hemispheric edema, according to the following formula: 1-vol of normal ipsilateral hemisphere/volume of total contralateral hemisphere) x 100 ​% [[30], [31], [32]].

### Shotgun proteomics

Total proteins (15 ​μg) were extracted from the residual brain tissue sections, mixed with lithium dodecyl sulfate lysis buffer (Thermo, Fisher Scientific Inc.), and incubated at 99 ​°C for 5 ​min. Proteins were then subjected to electrophoresis for 5 ​min at 200 ​V on a NuPAGE 4–12 ​% Bis-Tris gel in 1X MES/SDS (Thermo) running buffer. Gels were stained with SimplyBlue SafeStain (Thermo, Fisher Scientific Inc.) for 5 ​min, followed by an overnight wash in water with gentle agitation. The band containing the whole proteome from each sample was excised from the polyacrylamide gel and treated as previously described [33]. The proteins were in-gel proteolyzed with trypsin gold (Promega Corporation, USA) in the presence of 0.01 ​% Protease Max surfactant (Promega) at 50 ​°C for 60 ​min. One μL of the resulting peptide fraction (of the 50 ​μL total volume), corresponding to approximately 300 ​ng of peptide, was analyzed by liquid chromatography-tandem mass spectrometry (LC−MS/MS) with an Orbitrap Exploris 480 tandem mass spectrometer (Thermo Scientific) coupled to a Vanquish Neo UHPLC system, as previously described [34]. The peptides were loaded on a reverse-phase Acclaim PepMap 100C18 precolumn (5 ​μm, 100 ​Å, 300 ​μm i.d. ​× ​5 ​mm, Thermo Fisher) and then resolved on an Acclaim PepMap 100C18 column (3 ​μm, 100 ​Å, 75 ​μm i.d. ​× ​50 ​cm, Thermo Fisher) at a flow rate of 0.2 ​μL ​min^−1^ using a 90-min gradient (4–25 ​% B in 75 ​min, and 25–40 ​% B in 15 ​min), with 0.1 ​% HCOOH/100 ​% H_2_O as mobile phase A and 0.1 ​% HCOOH/100 ​% CH3CN as mobile phase B. The mass spectrometer was operated in data-dependent acquisition mode with a Top20 strategy, a scan range of 350–1800 ​*m*/*z*, and selection and fragmentation were performed using a 10 ​s dynamic exclusion time. Only ion precursors with a 2+ or 3+ charge were selected for HCD fragmentation. Peptides and proteins were identified from the dataset using the MaxQuant software version 1.6.6.0 [35] against the Rattus norvegicus (Norway rat) genome assembly mRatBN7.2. Carbamidomethylation of cysteine was set as a fixed modification, whereas variable modifications included oxidation of methionine, deamidation of asparagine and glutamine, and acetylation of protein N-terminal residue. Peptide tolerance, MS/MS fragment tolerance, minimum peptide length, and maximum number of missed cleavages were set to 20 ​ppm, 20 ​ppm, 7 residues, and 2, respectively. Peptide-to-spectrum matches and proteins were established at a false discovery rate (FDR) below 1 ​% with the option match between runs activated. The collapse of protein isoform replicates was based on the isoform with maximum abundance.

### Proteomics data interpretation

Differentially expressed proteins (DEPs) were identified using a modified version of the R/Bioconductor package for reproducibility-optimized statistical testing (ROTS) [36]. Specifically, the Maxquant proteomics LFQ data were Log_2_ transformed and normalized using variance stabilizing normalization (VSN) [37]. Pairwise comparisons were performed, and a modified T-statistic test was applied to rank the proteins according to the statistical evidence for significant differential expression between the three groups (Sham, HI ​+ ​Veh and BRT_002 treatments) in the ipsilateral and contralateral brain hemispheres of the male and female rats. Proteins were differentially abundant at p ​< ​0.05. Reproducibility plots and principal component analysis (PCA) were used to examine the quality of data separation between the various groups that were compared. Heatmaps were generated using unsupervised hierarchical clustering based on Ward linkage and Euclidean distance to examine the degree of proteomic profile separation across the groups. Stepwise regression statistical modeling was used to reduce further the marker set and identify the proteins whose abundance was significantly modified by treatment with BRT_002. Protein-protein interaction (PPI) network was constructed using GeneMANIA software with default parameters [38,39]. Proteins were annotated using Gene Ontology (GO) [40] the Kyoto Encyclopedia of Genes and Genomes (KEGG) database canonical pathways [40] from MSigDB, Reactome gene sets [41] and CORUM structural complexes [42] at a significance cut-off of p ​≤ ​0.01. Relevant enriched terms were selected based on the following criteria: enrichment factor ≥1.5, at least 3 overlapped proteins, and p ​≤ ​0.01. Additionally, the enrichment clustering for each comparison was further explored to select only the most abundant proteins and identify their potential functions in neonatal rats exposed to HI after treatment with BRT_002.

### Duolink proximity ligation assay

The proximity ligation assay was used to detect protein-protein interactions (PPIs) using the following key components: the PLA probe anti-rabbit PLUS (DUO92002), the PLA probe anti-mouse MINUS (DUO92004) and the detection reagent ORANGE (DUO92007). Paraffin-embedded slides were heated in a 60 ​°C oven for 1 ​h. Then, the slides were deparaffinized and rehydrated using the following steps: xylene twice for 5 ​min each, 100 ​% EtOH twice for 5 ​min each, 95 ​% EtOH twice for 3 ​min each, 70 ​% EtOH once for 3 ​min and distilled water twice for 3 ​min each. Antigen retrieval was performed in sodium citrate buffer at 95 ​°C for 20 ​min. The slides were washed 3 times after cooling in 10 ​mM TBS-T for 5 ​min each. A blocking solution (1 ​% bovine serum albumin; 5 ​% normal goat serum) was then applied at RT for 2 ​h. Samples were incubated overnight at 4 ​°C with the following primary antibodies: rabbit anti-synaptotagmin 5 (Bio-Techne, NBP1-69104) with mouse anti-Thioredoxin-2 (Thermo Scientific PA5-87276), rabbit polyclonal anti-AGRIN (Thermo Scientific PA5-103585), mouse anti-VEGFA (Protein Tech, 66828-1), rabbit polyclonal anti-Zyxin (Thermo Scientific PA5-68635), mouse monoclonal anti-Zyxin (Santa Cruz Biotechnology sc-293448), mouse monoclonal anti-VWF (Invitrogen MA541692) or rabbit polyclonal anti-Angiopoietin 1 (Thermo Scientific PA1-32150). The slides were incubated on the second day at 37 ​°C for 1 ​h in a preheated humidified chamber with a PLA mixture (the PLA probes PLUS and MINUS diluted 1/5 in the blocking solution and incubated for 20 ​min before application), after three washes with the blocking solution. The samples were incubated for 30 ​min at 37 ​°C with a ligation solution consisting of ligation stock and ligase diluted in high-purity water at ratios of 1/5 and 1/40, respectively, after having been exposed to two 5-min washes with wash buffer A [0.01 ​M Tris, 0.15 ​M NaCl, and 0.05 ​% Tween 20]. Cells were washed again with wash buffer A and incubated for 100 ​min with a mixture of amplification stock (1/5) and polymerase (1/80) diluted in high-purity water. Samples were then washed twice with 1 ​× ​wash buffer B [0.2 ​M Tris and 0.1 ​M NaCl] for 10 ​min and once in 0.01 ​× ​wash buffer B for 1 ​min. Finally, DAPI (0.1 ​μg/mL) was applied for 10 ​min, and the slides were washed 3 times in TBS-T for 5 ​min each and mounted in mounting medium (Sigma Aldrich, FluoromountTM aqueous mounting medium).

### mRNA extraction and RT‒qPCR

Total RNA was extracted with QIAzol® reagent and purified on RNeasy® Plus Universal Tissue Mini Kit columns (Qiagen, Courtaboeuf, France). Briefly, brain tissue samples were homogenized with 1 ​mL of QIAzol reagent using a Precellys 24 tissue homogenizer (Bertin technologies, France), and gDNA was eliminated. The mixtures were centrifuged at 12,000×*g* for 15 ​min at 4 ​°C, after the addition of 180 ​μL of chloroform. The resulting aqueous phases were mixed with 600 ​μL of 70 ​% ethanol and loaded onto RNeasy columns. Total RNA was washed and eluted with RNase-free water according to the manufacturer's protocol and stored at −80 ​°C. RNA concentrations were measured spectrophotometrically in a NanoDrop 2000c (Labtech France). cDNA was synthesized from 0.5 ​μg of total RNA with the RT^2^ HT First Strand kit (Qiagen, Courtaboeuf, France). Two μL of cDNA was mixed with 6.25 ​μL of iTaq™ Universal SYBR® Green Supermix (Bio-Rad, Marnes-la-Coquette, France) and 0.375 ​μL of a 10 ​μM primer mixture (Supplementary Table 1) and brought to 10 ​μL with ultrapure DNase-RNase-free distilled water for qPCR. qPCR was performed in a CFX96 Real-Time Detection System (Bio-Rad, Marnes-la-Coquette, France) with the following cycling conditions: denaturation at 95 ​°C for 10 ​min, 40 cycles of 15 ​s at 95 ​°C followed by 1 ​min at 60 ​°C, and a final step of 30 ​s at 72 ​°C. Cycle threshold (Ct) values of the target gene and housekeeping gene (hypoxanthine-guanine phosphoribosyl transferase, HPRT) were recorded, and gene expression was calculated as 2^−ΔCt^ (where ΔCt ​= ​Ct target – Ct HPRT).

### Mitochondrial respiration

Primary cortical neurons were prepared from embryonic day 14–16 pregnant C57BL/6 mice as previously described [43]. Briefly, cortices from embryos in a single litter were dissected, meninges removed, and tissue was pooled. Cortices were roughly chopped before incubation in 0.25 ​% trypsin/EDTA followed by trituration. Cells were pelleted by centrifugation, resuspended, and plated in a neurobasal medium supplemented with B27, 100 units/mL penicillin, 100 μg/mL streptomycin, 0.25 μg/mL amphotericin B, 300 μM glutamine and 25 μM 2-mercaptoethanol. Neurons were prepared and plated at a density of 30–50–70 ​× ​10^3^ ​cells/well. Treatments were performed on neurons grown for a minimum of 7 days *in vitro*, and all media were obtained from Invitrogen (Grand Island, NY, USA).

The growth medium was replaced with de-gassed, glucose-free, neurobasal-A medium (Invitrogen, Life Technologies Glasgow, UK) and culture plates were maintained at 37 ​°C in a 95 ​% N_2_/5 ​% CO_2_ environment for the duration of the oxygen-glucose deprivation (OGD) experiments. The medium was replaced with a standard neurobasal medium (containing additions as described above) and cultures returned to 5 ​% CO_2_/95 ​% air incubation, after the exposure to OGD. The medium was replaced in the control plates with standard media at the start of the OGD and replaced again after 90 ​min. BRT_002 was added at a final concentration of 10 ​μM for 24 ​h and then the BRT_002-containing medium was replaced by a glucose-free medium to initiate the OGD exposure.

Real-time measurements of oxygen consumption rates were performed on an XFe96 Seahorse extracellular flux analyzer (Seahorse Biosciences, North Billerica, MA, USA). The optimal seeding density and test compound concentrations were empirically determined before the initiation of the experiments. According to the methods described in the XFe96 extracellular flux analyzer user's manual (Seahorse Bioscience), preliminary studies were run with carbonyl cyanide-4-(trifluoromethoxy) phenylhydrazone (FCCP) to identify the optimal number of cells required to observe a sufficient shift in OCR. We verified the optimal working concentrations for each of the stimulating compounds used in the mitochondrial function analysis (oligomycin, FCCP, and rotenone), after the cell number had been determined. Cells were then plated onto XFe96 ​cell culture plates (Seahorse Biosciences, North Billerica, MA, USA) at a density of 50,000/well in 80 ​μL of DMEM (Sigma–Aldrich, St. Louis, MO, USA). Cells were allowed to adhere overnight in a 37 ​°C incubator with 5 ​% CO_2_. Two hundred μL of XF calibration media was added to the XF sensor cartridges and kept in a non-CO_2_ incubator for 24 ​h on the day before the experiment. XF sensor cartridges were loaded with test compounds and OCR was measured. The assay was performed by sequential injections of oligomycin (2 ​μM final concentration, blocks ATP synthase to assess respiration required for ATP turnover), FCCP (carbonyl cyanide 4-trifluoromethoxy-phenylhydrazone, 2 ​μM final concentration, a proton ionophore uncoupler inducing maximal respiration), and rotenone plus antimycin A (0.5 ​μM final concentration of each, which completely inhibits electron transport to measure non-mitochondrial respiration). Each step had three cycles; each cycle consisted of 3 ​min mixing, 2 ​min incubation and 3 ​min measurement. All experiments were run in three replicates with 3–4 samples per replicate. Cell counts were used to normalize OCR.

### Statistical analysis

All data were initially examined for outliers using the ROUT test [44] in GraphPad Prism software (GraphPad Software, San Diego, CA, USA). Statistical analyses were conducted with GraphPad Prism (GraphPad Prism version 9.4.0). The distributions of all experimental data were assessed for normality tests. If the data were normally distributed, the Sham, HI ​+ ​Veh and BRT_002-treated groups were compared by one-way analysis of variance (ANOVA) or two-way ANOVA with sex and treatment as factors, followed by Bonferroni correction as a post hoc test (*p ​< ​0.05*) to identify differences between the groups. If the data were not normally distributed, Kruskal‒Wallis ANOVA and the median tests were applied. Protein abundance was compared between two groups using the Wilcoxon test. A p-value <0.05 was considered to indicate statistical significance in all statistical tests.

## Results

### Synthesis of the valyl ester BRT_002

The compounds used in the current experiments are tri-substituted purine derivatives. Several trisubstituted purines and some structurally related heterocycles are kinase inhibitors [45,46]. These compounds bind to the kinase catalytic site by a hydrogen bond. The compounds BRT_001 and BRT_002 (Supplementary Fig. 1) that lack hydrogen on the N6 are not able to bind to kinases and, therefore, are devoid of kinase inhibitory activity [47]. The valyl ester, BRT_002 was preferred to its precursor BRT_001 in the present study. This approach has been previously used for the two antiviral medications acyclovir and ganciclovir. Their conversion into the corresponding valyl esters provided valacyclovir and valganciclovir [48] with improved bioavailability and enhanced therapeutic potential.

### Repeated high doses of the novel tri-substituted purine derivative, BRT_002, were nontoxic and well tolerated in wild-type adult male rats

The potential dosage ranges for the repeated dose study of BRT_002 were analyzed for possible toxicity before initiating a formal non-GLP toxicity study. Mortality was not identified even at the 100 ​mg/kg dose of BRT_002, and adverse clinical signs were not apparent. Macroscopic abnormalities were also not observed in the major organs at necropsy. Therefore, a dose of 100 ​mg/kg was selected for the 7-day fixed-dose non-GLP toxicity study.

The 7 day-fixed-dose study did not show obvious changes in body weight after administration of BRT_002 (Supplementary Fig. 2a). Additionally, BRT_002-treated animals did not show significant abnormalities in hematological, or coagulation profiles compared with the controls (p ​> ​0.05; Supplementary Table 1a). Other clinical variables including cholesterol concentrations did not show significant differences after treatment with BRT_002 compared with the control-treated animals (p ​> ​0.05; Supplementary Table 1b). The amount of BRT_001 within each organ was subsequently quantified by LC-MS/MS to determine its bioaccumulation because BRT_002 is rapidly cleaved to BRT-001. Significant BRT_001 accumulation was not observed in any organ after treatment with BRT_001 (Supplementary Table 2). All organs were recovered and weighed after sacrifice. The weights of the brain, spleen, liver, kidneys, or heart were not modified by daily administration of 100 ​mg/kg BRT_002 for 7 days (Supplementary Fig. 2b). These results indicate that the administration of BRT_002 ​at a dose of 100 ​mg/kg for seven days did not result in mortality, changes in clinical variables, food consumption, or accumulation in any major organ, suggesting that the drug has a wide safety profile.

### Penetration of BRT_002 in serum and brain of neonatal rats

Effective drug delivery was established by PK studies after BRT_002 had been administered to Sham and HI-exposed males and females via the IP route. This route of administration is routine for the delivery of drugs in studies of neonatal rats. Blood, ipsilateral and contralateral brain hemispheres were collected 1, 2, 6 and 24 ​h after the IP administration of 30 ​mg/kg of BRT_002. BRT_002 and BRT_001 were quantified by LC‒MS/MS.

BRT-001 levels were determined in the brain and plasma because BRT_002 is rapidly cleaved to BRT-001 in plasma. Systemic exposure of BRT_001 in the neonatal rats after the IP administration of 30 ​mg/kg of BRT_002 was approximately five times lower than that measured after the oral administration of 100 ​mg/kg to the adult rats, which did not result in adverse effects. The brain concentration of BRT_001 peaked at 1 ​h, the first time point, and then slowly declined to a few ng/mL at 24 ​h in both hemispheres of the sham-treated female and male neonatal rats after the IP injections (Supplementary Fig. 3a). The brain BRT_001 maximal concentrations were similar in sham-treated males (ipsilateral brain hemisphere: 667.6 ​ng/g and contralateral hemisphere: 609.2 ​ng/g) and females (ipsilateral hemisphere: 591.3 ​ng/g and contralateral hemisphere: 613 ​ng/g, Supplementary Fig. 3a). Similarly, the brain BRT_001 maximal concentrations of the HI treated group peaked 1 ​h after injection and were similar in the males (ipsilateral brain hemisphere: 553 ​ng/g and contralateral hemisphere: 926.8 ​ng/g) and females (ipsilateral brain hemisphere: 463.9 ​ng/g and contralateral hemisphere: 637.2 ​ng/g, Supplementary Fig. 3a). The concentrations of BRT_001 measured in serum were consistent with brain concentrations in all groups and sexes (Supplementary Fig. 3b).

The partition coefficient (the ratio of drug concentration in the brain to blood: Kp) was about one in both brain hemispheres of the males and females after exposure to HI (Supplementary Fig. 3c), whereas the Kp was between 0.6 and 0.7 in the sham-treated males and females, respectively. These findings reflect the ability of BRT_001 to penetrate the brain in both the Sham and HI-treated female and male neonatal rats. Consequently, these findings indicate that BRT_001 penetrates the brain parenchyma with maximum exposure occurring in the brain and serum approximately 1 ​h after IP BRT_002 administration.

### Brain pharmacokinetic modeling of BRT_001

The results of brain PK modeling of BRT_001 are summarized in Supplementary Table 3. The area under the curve (AUClast, ng.h/g) values for the ipsilateral and contralateral hemispheres were slightly larger in the sham male (2292 and 2528 ​ng.h/g, respectively) than in the sham female neonatal rats (1817 and 1763 ​ng.h/g, respectively), whereas the AUClast values in the ipsilateral and contralateral hemispheres were similar in the male and female rats after exposure to HI (2330 ng.h/g and 2522 ng.h/g versus 2854 ng.h/g and 3055 ng.h/g). The comparison of other pharmacokinetic parameters also did not reveal any differences in distribution between the cerebral hemispheres, between the two sexes, or between sham animals and animals exposed to HI. These findings suggest the absence of major sexual dimorphism in male and female neonatal rats with regard to the cerebral distribution of BRT_001 with and without exposure to HI.

### Treatment with BRT_002 attenuates ipsilateral infarct volume loss in neonatal rats after exposure to HI

**Fig. 1:**
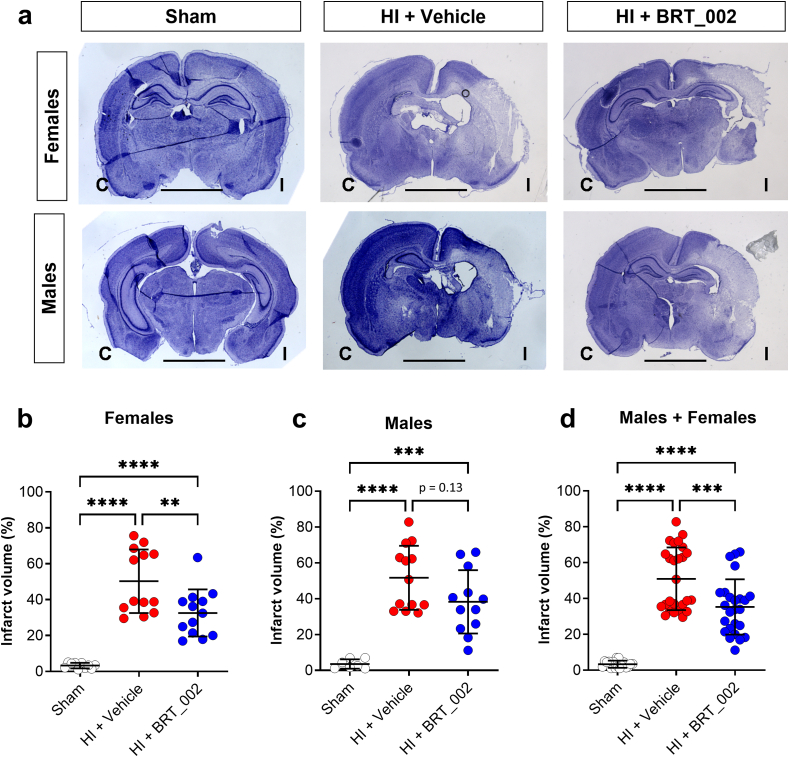
**BRT_002 attenuates brain injury in neonatal rats. a.** Representative images of brain sections stained with cresyl violet 72 ​h after exposure to HI in male and female rats in the sham, HI ​+ ​vehicle and HI ​+ ​BRT_002 groups. Scale bars ​= ​3 ​mm. Visual examination of the coronal images suggested differences between the groups. The female (**a**, top panel) and male (**a**, bottom panel) HI ​+ ​Veh group of neonatal rats appeared to exhibit decreased staining in the ipsilateral brain hemisphere compared with in the sham groups. I: Right hemisphere ​= ​ipsilateral to the HI (carotid ligation and hypoxia), C: left brain hemisphere ​= ​contralateral side. Percent infarct volume plotted on the y-axis for the sham, HI ​+ ​Vehicle and HI-BRT_002 treated groups: females (b), males (c) and males ​+ ​females (d). Sham designated by open circles, HI ​+ ​Vehicle designated by closed red circles, and HI ​+ ​BRT_002 (30 ​mg/kg) designated by closed blue circles. Sham: females, n ​= ​10; males, n ​= ​7; HI ​+ ​vehicle: females, n ​= ​13; males, n ​= ​13; HI ​+ ​BRT_002: females, n ​= ​13, males, n ​= ​12. Statistical analysis by one-way ANOVA and Bonferroni post hoc correction; ∗P ​< ​0.05; ∗∗P ​< ​0.01; ∗∗∗P ​< ​0.001; ∗∗∗∗ P ​< ​0.0001.

The effect of BRT_002 was determined in male and female neonatal rats after exposure to carotid ligation and 90 ​min of hypoxia. Inspection of the coronal brain images at P10 revealed that the HI ​+ ​Veh group exhibited increased ipsilateral hemispheric pallor compared with the sham control group in both the female (Fig. 1a top panel) and male (Fig. 1a, bottom panel) neonatal rats. Moreover, the BRT_002 treated group appeared to demonstrate reduced pallor compared with the HI ​+ ​Veh group in both male and female neonatal rats after exposure to HI.

Quantitative analysis of the percent infarct volume loss was performed to quantify the visual observations of the cresyl violet-stained brain sections from neonatal rats exposed to HI-related brain injury with and without BRT_002 treatment. The ipsilateral hemispheric infarct volume loss was substantially larger in the HI ​+ ​Veh females and males compared with the control sham group (females 50.26 ​± ​17.73 versus 3.27 ​± ​1.54, males: 51.67 ​± ​17.87 versus 3.56 ​± ​2.62; p ​< ​0.0001), respectively. Quantitative analysis of the percent infarct volume loss showed that the ipsilateral infarct volume was loss of 50.26 ​± ​17.73 ​% in the placebo (HI ​+ ​Veh) treated female group and was reduced to 32.55 ​± ​13.13 ​% after treatment with BRT_002 (Fig. 1b, p ​< ​0.01). The percent infarct volume loss was 51.67 ​± ​17.87 ​% in the placebo (HI ​+ ​Veh) treated male group and 38.28 ​± ​17.70 ​% after treatment with BRT_002 (Fig. 1c, p ​= ​0.1322). The ipsilateral hemispheric infarct volumes were also larger (50.96 ​± ​17.46 ​%) in the HI ​+ ​Veh for the entire cohort of the males plus females compared (p ​< ​0.001) with the BRT_002 treated cohort (35.30 ​± ​15.44 ​%, Fig. 1d, p ​< ​0.001). Treatment with BRT_002 reduced the infarct volumes in the females by 35.22 ​± ​26.12 ​%, p ​< ​0.01, and in the entire cohort of males plus females by 30.73 ​± ​30.29 ​%, p ​< ​0.001. However, the 25.91 ​± ​34.25 ​% reduction in the infarct volumes after treatment with BRT_002 in the males was not statistically significant (p ​= ​0.1322). Nonetheless, sex did not have a major effect, and treatment-related sex interactions were not detected (Fig. 1b–d, Tukey's HSD, all p ​> ​0.05). Taken together, these findings can be interpreted to suggest that treatment with three doses of BRT_002 after exposure to HI attenuates neuronal loss/damage and preserves the integrity of brain tissue.

### BRT_002 improves locomotor activity in neonatal rats after exposure to HI

Locomotor tests were conducted to evaluate the potential impact of treatment with BRT_002 on early motor outcomes of the neonatal rats after exposure to HI. Walking acquisition was significantly delayed after exposure to HI. All Sham treated female animals were able to walk on P10 compared to a small percentage (10 ​%) in the HI ​+ ​PL group. Remarkably, 20 ​% of the female HI ​+ ​PL pups were still crawling on P10. In contrast, almost all (93.75 ​%) of the HI ​+ ​BRT_002 treated female animals were able to walk on P10. The majority (71.43 ​%) of the Sham treated male pups were able to walk on P10 compared with 33.33 ​% of the HI ​+ ​PL and all the BRT_002 treated male pups. Crawling was also still present in 4.76 ​% of the male pups on P10 after exposure to HI. These findings suggest that exposure to HI appears to delay the ability to walk on P10 in both male and female pups and that treatment with BRT_002 improves the ability to walk in both sexes (Supplementary Fig. 4).

The righting reflex was used to test early motor coordination and negative geotaxis to determine vestibular reflexes, strength, and coordination in neonatal rats after exposure to HI related brain injury. The righting reflex was not able to detect differences in the male or female pups on P10 (Sham/HI ​+ ​PL/HI ​+ ​BRT_002) and the negative geotaxis test did not identify differences on P8, P9, or P10 after exposure to HI related brain injury (Sham/HI ​+ ​PL/HI ​+ ​BRT_002). These results can be interpreted to suggest that these tests are not sufficiently sensitive to detect behavioral disparities among the different groups after carotid ligation and exposure to 8 ​% FiO_2_ for 90 ​min. Consequently, the potential effects of treatment with BRT_002 cannot be discerned by examining these tests.

### Proteomic analysis revealed that BRT_002 treatment results in a unique protein profile in neonatal rats after exposure to HI-related brain injury

Proteomic profiles were explored in the different groups to decipher the molecular mechanisms fundamental to the preservation of brain tissue and the reduction in neuronal damage in neonatal rats treated with BRT_002 after exposure to HI. An in-depth exploration of the protein profiles was conducted in the ipsilateral and contralateral brain hemispheres in the BRT_002-treated male and female neonatal rats after exposure to HI. The profiles were analyzed by label-free shotgun proteomics on 60 biological samples. The distribution of biological replicates among the three groups (Sham, HI ​+ ​Veh and HI ​+ ​BRT_002 groups) is summarized in Supplementary Table 5. A total of 3724 proteins were identified and their abundance monitored among the 60 biological samples. Given a possible difference in the therapeutic effects of BRT_002 between males and females, differential protein expression analysis was performed for each comparison of ipsilateral and contralateral brain hemispheres from neonatal rats in the HI ​+ ​BRT_002 and HI ​+ ​Veh groups after exposure to HI, with and without separation by sex. Significantly up- and downregulated proteins (p ​< ​0.05) between the studied groups are shown in Supplementary Table 6 for the ten resulting comparisons. Principal component analysis (PCA) assessed favorably the degree and quality of data separation between the different comparisons in the Sham, HI ​+ ​Vehicle and HI ​+ ​BRT_002 groups of neonatal rats (Supplementary Fig. 5). As expected, all comparisons of differentially expressed proteins (DEPs) in the ipsilateral versus contralateral brain hemispheres of animals in the HI-BRT_002 and HI ​+ ​Veh groups with and without separation by sex identified unique abundant proteins in each specific group. This further suggests that BRT_002 has distinct effects in males versus females and the ipsilateral versus contralateral brain hemispheres. Functional enrichment analysis of the DEPs identified by each described comparison of the HI ​+ ​BRT_002 and HI ​+ ​Veh groups of neonatal rats was determined, and the results are presented in Supplementary Fig. 6.

### Proteomic analysis identified agrin and zyxin in the ipsilateral and contralateral hemispheres of neonatal rats treated with BRT_002 after exposure to HI-related brain injury

**Fig. 2:**
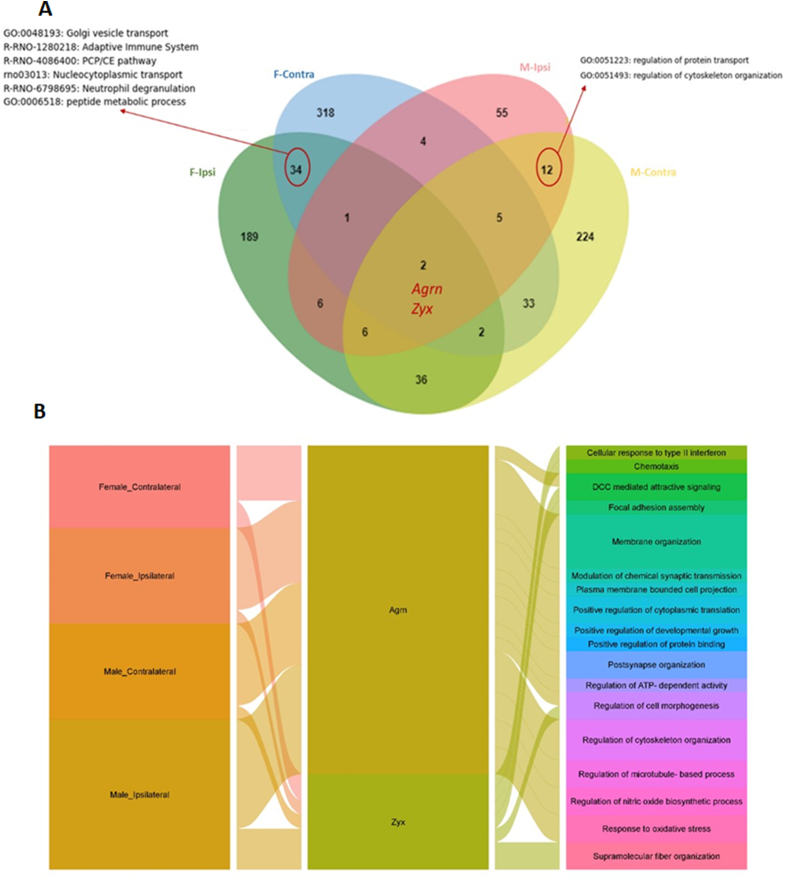
**Proteomic analysis revealed that treatment with BRT_002 results in unique protein expressions in the ipsilateral and contralateral sides of the brain in male and female neonatal rats after exposure to HI.** a) Comparisons of the differentially expressed proteins (DEPs) in the HI ​+ ​BRT_002 vs. HI ​+ ​Veh groups of males and females and separately for the ipsilateral and contralateral brain hemispheres. F: female; M: male; Ipsi: ipsilateral; Contra: contralateral. b) Alluvial plot of agrin- and zyxin-related pathways among the different comparisons between the HI ​+ ​BRT_002 and HI ​+ ​Veh groups of neonatal rats. The left column shows the different comparisons between the HI ​+ ​BRT_002 and HI ​+ ​Veh groups of neonatal rats, the right column shows the related pathways, and the edges represent their relationship with agrin and zyxin. A larger edge width represents pathway-related systems.

Comparison of DEPs in the ipsilateral and contralateral brain hemispheres of HI ​+ ​BRT_002 and HI ​+ ​Veh males and females identified 34 proteins common to both brain hemispheres of the female BRT_002-treated neonatal rats. These proteins were found to be mainly involved in the adaptative immune response, Golgi vesicle transport, nucleoplasmic transport, the planar cell polarity pathway (PCP)/CE (a process by which a tissue narrows along one axis and lengthens along a perpendicular one) pathway, and peptide metabolic processes (Fig. 2a, Supplementary Table 7). Twelve proteins common to both brain hemispheres were identified. They are mainly involved in the regulation of protein transport and organization of the cytoskeleton (Fig. 2a–Supplementary Table 8).

Integration of the comparative protein profiles in both hemispheres of male and female BRT_002-treated neonatal rats after exposure to HI identified two common proteins, specifically Agrin (gene ID: *Agrn*) and Zyxin (gene ID: *Zyx*). Interestingly, both proteins are involved in overlapping pathways and are pivotal in the regulation of complex components that associate the extracellular matrix and the cytoskeleton with brain injury (Fig. 2b).

### Treatment with BRT_002 attenuates alterations in mitochondrial foci, neurotransmitter expression, calcium sensors, and markers of neuronal cell death in the brains of neonatal rats after exposure to HI

**Fig. 3:**
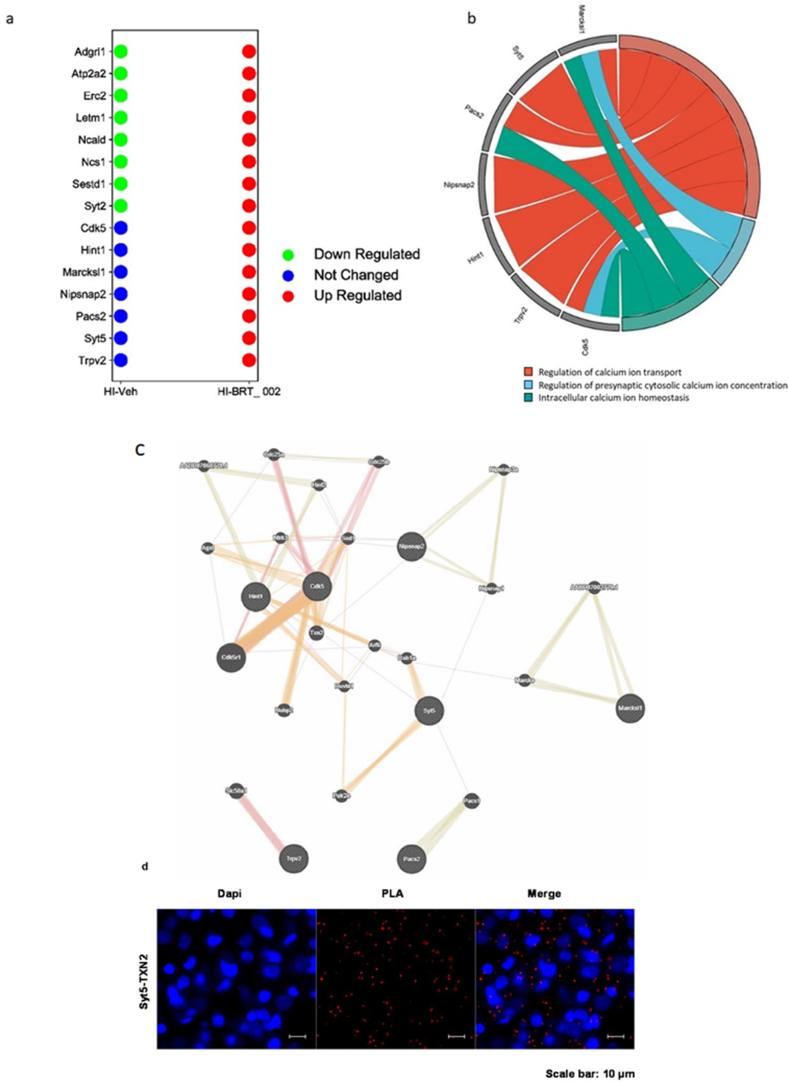
**BRT_002 modulates neurotransmitters and calcium sensors, which were selected from the comparison of the HI ​+ ​BRT_ 002 and HI ​+ ​Veh groups of neonatal rats after exposure to moderate HI. a**) Dots plot showing the protein signatures of corrected protein expression that were downregulated (green dots) or not changed (blue dots) compared to the sham group. Red dots indicate upregulated proteins after BRT_002 treatment compared with the HI ​+ ​Veh group. **b)** Pathways related to BRT_002-induced neurotransmitters and calcium sensors after exposure to HI. Functional enrichment analysis of BRT_002-induced neurotransmitters and calcium sensors resulted from the comparison of the HI ​+ ​BRT_ 002 and HI ​+ ​Veh groups of neonatal rats after exposure to HI. **c)** Protein‒protein interaction network of neurotransmitters and calcium sensors, whose expression was induced by BRT_002 treatment of neonatal rats after exposure to HI. The connections between these proteins were examined based on physical interactions, co-expression, predicted colocalization, common pathways, and shared protein domains. **d)** A representative Duolink Proximity Ligation Assay showing the interaction between Syt5 and Txn2 in BRT_002 treated male and female neonatal rats.

An in-depth analysis of the proteomic profiles revealed distinct signatures of proteins involved in the modulation of neurotransmitters and calcium sensor activity in the HI ​+ ​BRT_ 002 compared with the HI ​+ ​Veh treated neonatal rats. The most important finding was that 15 DEPs were significantly upregulated by treatment with BRT_002 compared with the vehicle-treated group after exposure to HI. The abundance of DEPs was further examined in the HI ​+ ​Veh group to elucidate the effects of treatment of the neonatal rats exposed to HI with BRT_002 compared with the sham control group. This analysis revealed that Adgrl1, Atp2a2, Erc2, Letm1, Ncald, Ncs1, Sestd1, and Syt2 were overrepresented, whereas Cdk5, Hint1, Marcksl1, Nipsnap2, Pacs2, Syt5, and Trpv2 were induced after treatment with BRT_002 in rats that were exposed to HI (Fig. 3a). These DEPs are neurotransmitters or calcium sensors that are associated with the regulation of mitochondrial membranes (e.g., Phosphofurin Acidic Cluster Sorting Protein 2, Pacs2), developmental and cellular mitochondrial respiration (e.g., Leucine Zipper And EF-Hand Containing Transmembrane Protein 1, Letm1), dendrite arborescence (e.g., SEC14 and Spectrin Domain Containing 1, Sestd1), synaptic transmission (e.g., Synaptotagmin 5, Syt5; Synaptotagmin 2, Syt2; SEC14 and Spectrin Domain Containing 1, Sestd1; and neuronal calcium Sensor 1, Ncs1), and regulation of neuroinflammation (e.g., Neurocalcin Delta, Ncald).

Functional enrichment analysis revealed that the major pathways related to the neurotransmitters and calcium sensors, whose production was increased by treatment with BRT_002 after exposure to HI, are involved in the regulation of calcium ion transport, regulation of presynaptic cytosolic calcium ion concentrations, and intracellular calcium ion homeostasis (Fig. 3b). Interestingly, Syt5 was the only commonly upregulated protein in the HI ​+ ​BRT_ 002 compared to HI ​+ ​Veh groups among all subgroups, when both male and female animals and the ipsilateral and contralateral brain hemispheres were included together in the analysis. This distinction reflects its commonality across all studied conditions of HI ​+ ​BRT_002 compared to the HI ​+ ​Veh groups, suggesting a potential role for this protein in the regulation of neuroprotection and neuronal activity mediated by BRT_002 after exposure to HI regardless of the animal sex and the damaged brain hemisphere.

A protein-protein interaction (PPI) network was constructed to identify protein networks that facilitate the ability of BRT_002 to promote neuroprotection and reflect interactions between neurotransmitters and calcium sensors, whose production was induced by BRT_ 002. These factors included Cdk5, Hint1, Marcksl1, Nipsnap2, Pacs2, Syt5, and Trpv2, (Fig. 3c). Remarkably, Syt5, Cdk5, Hint1, Marcksl1, Nipsnap2 and Pacs2 all interact with multiple markers. This suggests the possibility of interactions between these proteins through associations with predicted markers from similar families. The Syt5 subnetwork showed close associations with key markers of mitochondrial/respiratory function including Pacs1, Txn2, and Rab1a. This suggests that Syt5 could be a protein driver of neuroprotection facilitated by mitochondria. Txn2 is a key mitochondrial protein involved in the regulation of oxidative stress. The potential interactions between Txn2 and Syt5 were further investigated by performing a Duolink proximity ligation assay (PLA) which detects protein-protein interactions or protein modifications. Fig. 3d shows the immunofluorescence signal (red PLA dots) resulting from simultaneous immunostaining for both Syt5 and Txn2 with specific antibodies. Hence, there appears to be an *in-situ* close association between Syt5 and Txn2.

**Fig. 4:**
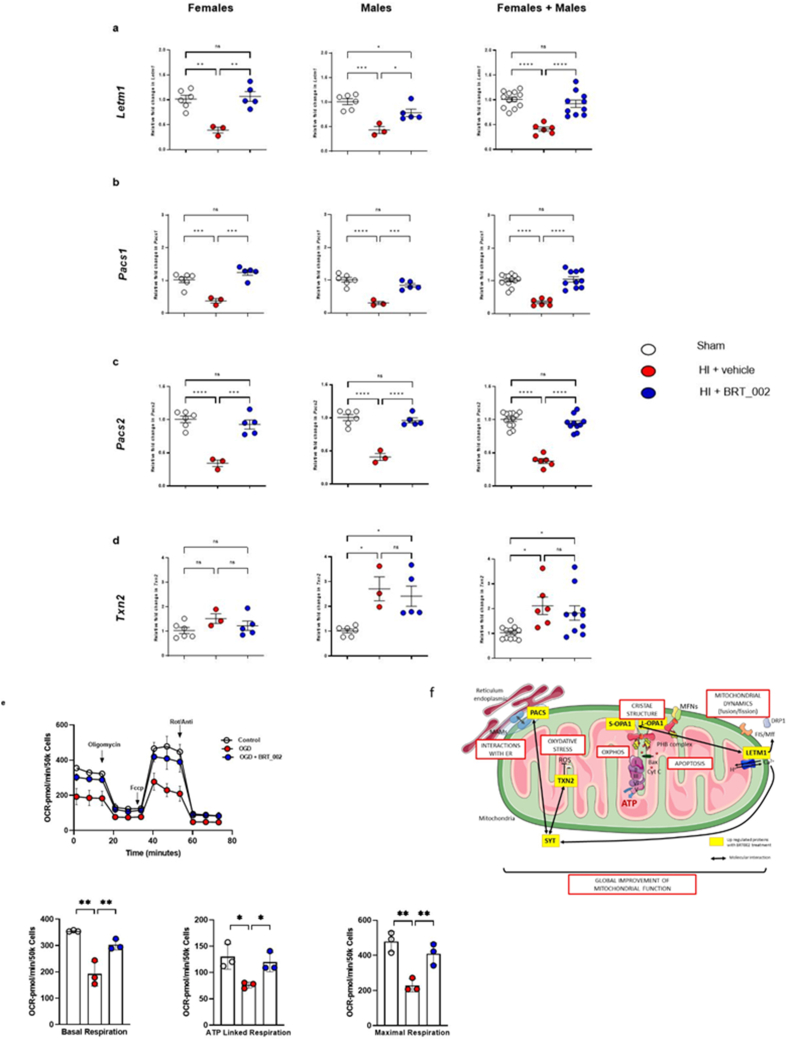
**BRT_002 modulates the transcriptional expression of key mitochondrial components in the ipsilateral hemisphere of female and male neonatal rats exposed to moderate HI and improves mitochondria function in HI ​+ ​Veh group 72 ​h after exposure to HI related brain injury**. **(a)***Letm1*, **(b)***Pacs1*, **(c)***Pacs2*, and **(d)***Txn2*. Data for the ipsilateral hemisphere in females (sham, n ​= ​6; HI ​+ ​Vehicle, n ​= ​3; and HI ​+ ​BRT_002, n ​= ​5), males (sham, n ​= ​6; HI ​+ ​Vehicle, n ​= ​3; and HI ​+ ​BRT_002, n ​= ​5) and males ​+ ​females (sham, n ​= ​12; HI ​+ ​Vehicle, n ​= ​6; and HI ​+ ​BRT_002, n ​= ​10) compared to those in the Sham and HI ​+ ​Vehicle groups. Sham designated by open circles, HI Vehicle designated by closed red circles, and HI ​+ ​BRT_002 (30 ​mg/kg) designated by closed blue circles. ∗P ​< ​0.05; ∗∗P ​< ​0.01; ∗∗∗P ​< ​0.001; ∗∗∗∗ P ​< ​0.0001, one-way ANOVA, Tukey's multiple comparison test. (e) Regulation of mitochondrial function. Seahorse measurements in control, OGD exposed (60 ​min) and BRT_002 (10 ​μM for 24 ​h) ​+ ​OGD exposed primary neuronal cells. Data represent three independent experiments expressed as mean ​± ​SD (n ​= ​3). Statistical analysis by one-way ANOVA with ∗p ​< ​0.05, ∗∗p ​< ​0.01. Bar graphs represent individual data points. **(f)** Schematic diagram summarizing the molecular interactions regulated by BRT_002 in mitochondria.

On the other hand, qPCR experiments revealed increased mRNA expression of four genes, which encode proteins concerned with mitochondrial function including *Letm1* (Fig. 4a), *Pacs1* (Fig. 4b), *Pacs2* (Fig. 4c), and *Txn2* (Fig. 4d) in the BRT_002-treated female and male compared with the vehicle-treated neonatal rats after exposure to HI. Taken together, these findings suggest that changes in key functional mitochondrial components are associated with the positive regulation of Syt5 by BRT_002, which could influence neuronal function and neuroprotection. Consequently, it may be assumed that BRT_002 most likely modulates proteins involved in the regulation of mitochondrial function after exposure to HI.

Oxygen Consumption rate (OCR) in primary mouse neurons exposed to OGD and BRT_002 showed that basal OCR and ATP-linked OCR were significantly increased in neurons after exposure to 10 ​μM of BRT_002 for 24 ​h compared with vehicle-treated control neurons (Fig. 4e). Carbonyl cyanide *p*-trifluoromethoxyphenylhydrazone (FCCP)-induced maximal OCR and spare respiratory capacity (SRC) decreased, whereas leak-driven OCR significantly increased after exposure to 10 ​μM of BRT_002. These results suggest that one of the major roles of BRT_002 could be the regulation of mitochondrial activity in neurons and, consequently, providing beneficial neuroprotective effects (Fig. 4f). Fig. 4f contains potential molecular interactions between the mitochondrial proteins that are upregulated by BRT_002.

**Fig. 5:**
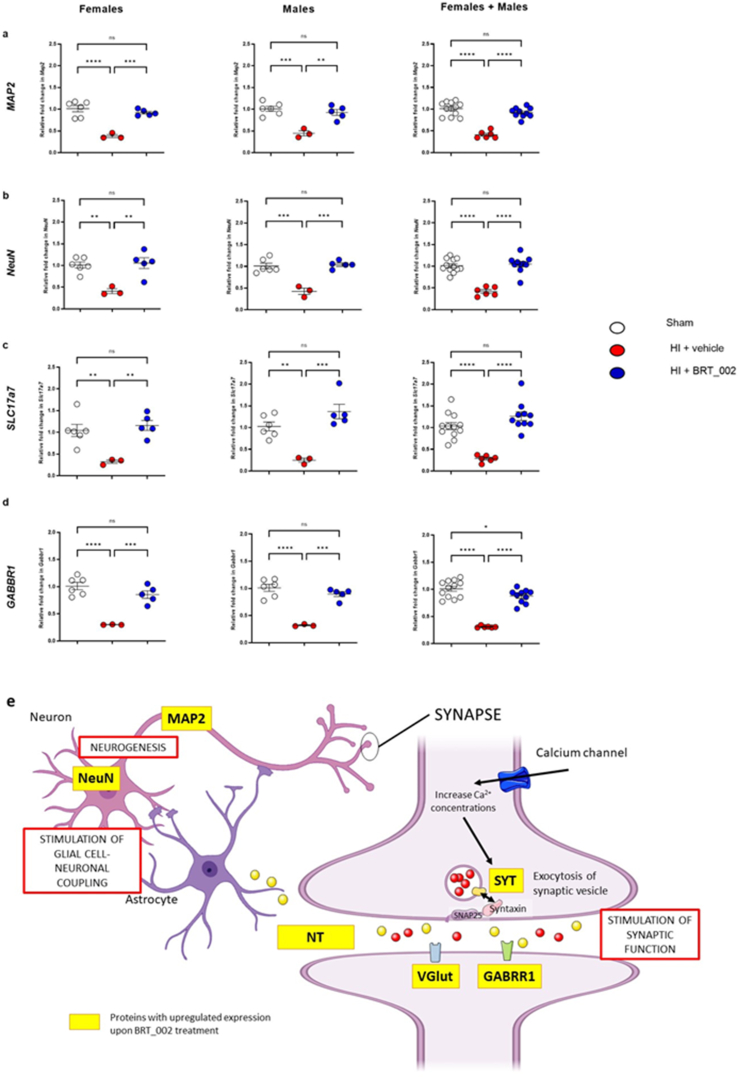
**Transcriptional expression of key synaptic markers in the ipsilateral brain hemisphere of female and male neonatal rats in the Sham, HI ​+ ​Veh and HI ​+ BRT_002 groups 72 ​h after exposure to HI related brain injury.** (a) *MAP2*, (b) *NeuN*, (c) *SLC17a7*, (d) and *GABBR1* in the ipsilateral hemisphere. Data for females (sham, n ​= ​6; HI ​+ ​Vehicle, n ​= ​3; and HI ​+ ​BRT_002, n ​= ​5), males (sham, n ​= ​6; HI ​+ ​Vehicle, n ​= ​3; and HI ​+ ​BRT_002, n ​= ​5) and males ​+ ​females (sham, n ​= ​12; HI ​+ ​Vehicle, n ​= ​6; and HI ​+ ​BRT_002, n ​= ​10) were compared to those for the control and vehicle groups. ∗P ​< ​0.05; ∗∗P ​< ​0.01; ∗∗∗P ​< ​0.001; ∗∗∗∗ P ​< ​0.0001, One-way ANOVA, Tukey's multiple comparison test**. e) Schematic representation of the interactions between synaptic proteins regulated by BRT_002 treatment. NT: Neurotransmitter**.

These results are consistent with the findings suggesting that exposure to HI reduced mRNA expression for markers of neuronal cells and that BRT_002 increased the mRNA expression of *Map2* (Fig. 5a), *Neun* (Fig. 5b), *Slc17a7* (Fig. 5c) and the GABAergic marker, *Gabrr1* (Fig. 5d) in neonatal rats after exposure to HI. Taken together, these findings strongly suggest that treatment with BRT_002 after exposure to HI prevents neuronal death in the ipsilateral brain hemisphere in both female and male neonatal rats in part by its effects on these neuronal constituents (Fig. 5e). Fig. 5e contains a schematic representation of the proteins that are upregulated at the neuronal synapse by treatment with BRT_002 after exposure to HI, thereby facilitating some of the neuroprotection observed after treatment with BRT_002.

### Potential interactions between Syt5, agrin and zyxin in brain tissue repair and vascular stability

**Fig. 6:**
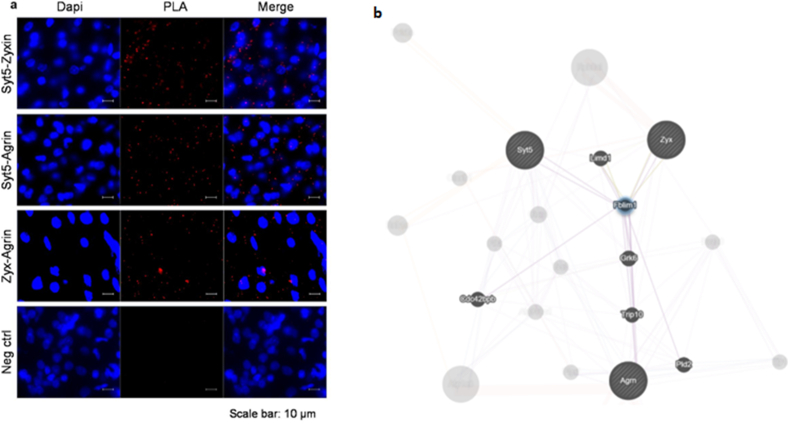
**Association between Syt5, Agrin and Zyxin**. (**a**) Duolink Proximity Ligation Assay (PLA) demonstrating in situ interactions between Syt5-Zyxin, SYt5-Agrin, and between Zyxin-Agrin. (**b**) In silico protein-protein interactions (PPI) network suggesting indirect connections between Syt5, Agrin, Zyxin and Filain Binding LIM protein 1 (Fblim1).

*In situ* PLA was performed with specific antibodies against Syt5, Agrin and Zyxin proteins to demonstrate further interactions between the three key proteins identified that have important roles in the potential neuroprotective effects of BRT_002 after exposure to HI-related brain injury. Syt5 appears to interact with Agrin and Zyxin at the endogenous protein level (at distances <40 ​nm, Fig. 6a). These findings are consistent with the involvement of Agrin and Zyxin in overlapping pathways, along with their potential participation in brain tissue repair and/or attenuation of the HI-related brain injury. Moreover, the *in-silico* PPI network suggests that there are prominent indirect interactions between Syt5, Agrin and Zyxin involving mainly Filamin Binding LIM Protein 1 (Fblim1), which was thus identified as an important intermediate participant in these interactions. This suggests that these proteins are functionally related or controlled by the same transcriptional regulatory pathways (Fig. 6b).

**Fig. 7:**
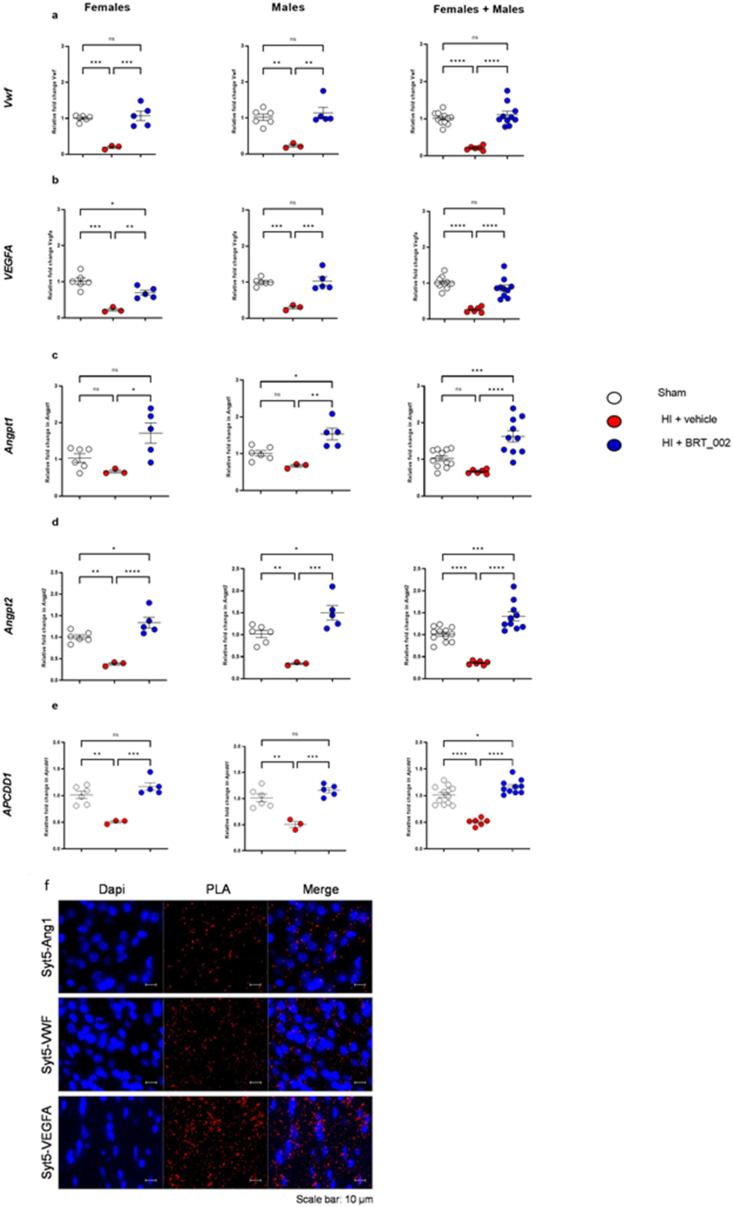
**BRT_002 facilitates transcriptional upregulation expression of key markers of brain capillaries related to agrin and zyxin in the ipsilateral hemisphere of female and male neonatal rats after exposure to moderate HI. a**) mRNA expression of *Von Willebrand Factor (Vwf)*; **b**) *VEGFA*, **c)** Angiopoietin 1 (*Angpt1)*, **d)** Angiopoietin 2 (*Angpt2)*, and e) *APCDD1*. Sham designated by open circles, HI Vehicle designated by closed red circles, and HI ​+ ​BRT_002 (30 ​mg/kg) designated by closed blue circles. Data for the ipsilateral hemisphere of females (sham, n ​= ​6; HI ​+ ​Vehicle, n ​= ​3; and HI ​+ ​BRT_002, n ​= ​5), males (sham, n ​= ​6; HI ​+ ​Vehicle, n ​= ​3; and HI ​+ ​BRT_002, n ​= ​5) and males ​+ ​females (sham, n ​= ​12; HI ​+ ​Vehicle, n ​= ​6; and HI ​+ ​BRT_002, n ​= ​10) were compared to those of the control and vehicle groups. ∗P ​< ​0.05; ∗∗P ​< ​0.01; ∗∗∗P ​< ​0.001; ∗∗∗∗ P ​< ​0.0001 one-way ANOVA, Tukey's multiple comparison test. (**f**) PLA assay showing in situ interactions between Syt5-VWF, Syt5-VEGFA and Syt5-Ang1.

Zyxin has been reported to export VWF [49]. VEGFA is involved in VWF exocytosis for brain tissue repair. Previous reports have suggested that VWF is secreted locally by cellular activation after vascular brain injury for brain repair [50,51]. Therefore, we performed RT‒qPCR for *Vwf* transcripts on samples from the ipsilateral brain hemisphere of BRT_002-treated female and male neonatal rats after exposure to HI. Importantly, we found that BRT_002 treatment of rats after exposure to HI strongly increased *Vwf* mRNA expression 72 ​h after injury (Fig. 7a) in the female (p ​< ​0.001) and male rats (p ​< ​0.01), and in the total group of males plus females (p ​< ​0.0001). Additionally, qRT-PCR revealed a significant increase in the *Vegfa* gene expression in the brain endothelial cells (Fig. 7b) of the HI-exposed BRT_002 treated group compared with the HI-Veh group of females (p ​< ​0.01), males (p ​< ​0.001), and the total group of males plus females (p ​< ​0.0001). Other factors, such as angiopoietins, have been reported to exhibit crosstalk with VEGFA, modulating its effects. Angiopoietin-1 (Ang1) and angiopoietin-2 (Ang2) compete for binding to Tie-2, whereas Ang1 promotes blood-brain barrier (BBB) stabilization [52]. Therefore, the mRNA expressions of *Ang1* and *Ang2* were examined. The mRNA expression of *Ang1* (Fig. 7c) in the ipsilateral hemisphere was significantly higher in the HI-BRT_002 treated compared with the HI-Veh groups of females (p ​< ​0.05), males (p ​< ​0.01), and in the total group of female plus male neonatal rats (p ​< ​0.0001). In addition, the expression of *Ang2* (Fig. 7d) in the ipsilateral hemisphere was significantly higher in the HI ​+ ​BRT_002-treated compared with the HI-Veh groups of female (p ​< ​0.0001), male (p ​< ​0.001), and in the total group of male plus female neonatal rats (p ​< ​0.0001). The role of Ang1 in the stabilization of the BBB is underscored by the elevated mRNA expression of *Apccd1*-CNS in the HI-BRT_002 compared with the HI-Veh female (p ​< ​0.001) and male (p ​< ​0.001) neonatal rats (Fig. 7e). These findings suggest that BRT_002 affects Zyxin expression and probably the crosstalk between Vegfa, Vwf*,* Ang1, Ang2 and Apcdd1 to enhance vascular stability after exposure to HI-related brain injury.

In addition, a Duolink PLA was used to examine potential interactions between Zyxin, Agrin, and Syt5. The PLA analysis unexpectedly exhibited *in-situ* interactions between Syt5, Vwf, Vegfa, and Ang1 (Fig. 7f, immunofluorescence signal, red dots on PLA) upon immunostaining for different proteins using specific antibodies. PLA dots were not observed when cells were stained with either the Vegfa, Vwf, or Ang1 antibodies alone or the Syt5 antibody alone. Taken together, these findings suggest that Syt5 interacts with major participants required for attenuation of injury processes, neuroprotection and/or parenchymal brain repair through Fblim1 that include Agrin, Zyxin, Vegfa, Vwf and mitochondrial targets.

### BRT_002 attenuates neuroinflammation in neonatal rats after exposure to HI

Neuroinflammation is an important element in the evolution of HI-related brain injury [[53], [54], [55]]. Therefore, astrocytic and microglial mRNA expression were examined as markers of neuroinflammation after treatment of HI-related brain injury with BRT_002. Glial fibrillary acidic protein (*GFAP*) gene expression was quantified after HI to reflect astroglioses (Supplementary Fig. 7a). Ipsilateral mRNA expression of *GFAP* was not significantly lower in the HI-BRT_002 than in the vehicle-treated females after exposure to HI. However, the male neonatal rats exhibited substantial increases (p ​< ​0.01) in *GFAP* mRNA expression in the vehicle-treated rats along with decreases (p ​< ​0.05) in *GFAP* after treatment with BRT_002.

Microglia are the first cells to become activated after exposure to HI insults [[56], [57], [58]] and serve to attenuate damage to the brain parenchyma. Therefore, the effect of treatment with BRT_002 was determined on ionized calcium-binding adaptor molecule 1 (*Iba-1*) mRNA expression as a reflection of the amount of microglia that could be present after exposure to HI. Gene expression was quantified at 24 (Supplementary Fig. 7b) after exposure to HI. *Iba-*1 mRNA expression was significantly increased in the HI-Veh group 24 ​h after exposure to HI (p ​< ​0.0001) but was not significantly reduced by treatment with BRT_002 (p ​= ​0.0108). However, the *Iba-*1 mRNA expression was higher in the vehicle than the HI-BRT_002-treated males after exposure to HI. Similarly, *Iba-1* expression remained significantly higher 72 ​h exposure to HI in the total cohort of male plus female neonatal rats in the HI-Veh (p ​= ​0.0161) and HI-BRT_002 groups (p ​= ​0.0412) compared with the sham group. These findings tend to suggest that BRT_002 also attenuates neuroinflammation in neonatal rats after HI.

## Discussion

The primary objective of the current study was to determine the ability of treatment with a novel tri-substituted purine-derivative (BRT_002) to attenuate neuropathological brain injury after exposure to HI in neonatal rats, and to determine some of the potential molecular mechanism(s) fundamental to its neuroprotective properties.

BRT_002 was developed as a potential drug to treat brain injury resulting from HI in neonates. We had previously shown that the parent compound, is distributed to the brain, can cross the BBB *in vitro,* and can rescue cognitive deficits associated with aging in mice [59]. In this context, treatment with the parent compound also prevented impairment of neurogenesis, enhanced synaptic function, reduced neuroinflammation by decreasing interleukin-1β expression, and activation of astrocytes, and microglia. Many of these processes are also similar to those that are fundamental to the development of HI-related brain injury in newborns [60]. Based upon our previous findings, we developed a modified BRT_002 compound for use in neonatal subjects because it lacks hydrogen on the N6 which renders it unable to bind to kinases and, therefore, is devoid of kinase inhibitory activity [47]. A similar approach has been previously used for two antiviral medications, acyclovir and ganciclovir. Their conversion into the corresponding valyl esters valacyclovir and valganciclovir [48] resulted in improved bioavailability and enhanced therapeutic effects.

BRT_002 was well tolerated in adult and neonatal rats and significant adverse events were not detected. The pharmacokinetic analysis demonstrated that IP administration of BRT_002 immediately after HI resulted in substantial systemic and brain concentrations of BRT_002 in sham control and in neonatal rats exposed to HI. Exposure to HI was associated with similar pharmacokinetic parameters in the male and female neonatal rats. Consequently, the preclinical safety and PK studies represent favorable initial findings to enable the use of BRT_002 in potential regulatory toxicology studies and possibly for a future phase I clinical safety and phase II-controlled trials.

The major findings of our study were that treatment with BRT_002 exhibited significant neuropathological protection in neonatal rats after exposure to HI and that the neuroprotective efficacy of BRT_002 was based upon dynamic interactions between multiple components of biological systems critical for the treatment of HI-related brain injury. The principal fundamental basis for neuroprotective effects of BRT_002 are most likely related to its effects on multiple aspects of brain injury that result from exposure to HI [60] including, neuronal preservation, mitochondrial function, potential beneficial effects on the microvasculature, and attenuation of inflammation along with possible interactions among these multiple factors. The ability of BRT_002 to target multiple components of HI-related brain injury is most likely fundamental to its neuroprotective efficacy. Furthermore, the novel tri-substituted purine derivative, BRT_002 could also facilitate crosstalk between the microvasculature, mitochondrial, and neuronal compartments to improve neuronal plasticity.

The results of our study provide *in vivo* evidence suggesting the potential for this novel substituted purine derivative to improve neuropathological outcomes after exposure to HI related brain injury in neonatal subjects. Nonetheless, it is also important to demonstrate that BRT_002 has beneficial effects on neurobehavioral outcomes. Therefore, we investigated the ability of treatment with BRT_002 to improve early motor outcomes in neonatal rats. Treatment with BRT_002 was shown to ameliorate deficits in the ability of the male and female neonatal rats to walk. However, similar to our previous report exposure to carotid ligation and 8 ​% FiO_2_ for 90 ​min did not detect abnormalities in the vehicle-treated animals on the righting reflex or negative geotaxis tests [61]. Consequently, these tests cannot be used to discern the effects of BRT_002 on these neurobehavioral outcomes. These findings are also consistent with previous work suggesting that exposure to longer durations of exposure to HI might be required to show more robust learning deficits [62,63].

**Fig. 8:**
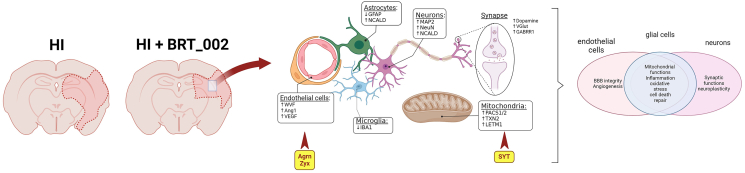
**Schematic diagram summarizing the major neuroprotective effects of BRT-002 on HI related brain injury in the neonatal rats.** BRT-002 exerts multiple beneficial neuroprotective effects in neonatal rats exposed to HI related brain injury. BRT-002 attenuates HI related brain injury as suggested by relative brain tissue preservation (Fig. 1). Some of the mechanisms of the neuroprotection potentially include potential beneficial effects on mitochondrial function as suggested by the *in vitro* OGD studies (Fig. 4), potential neuronal preservation (Fig. 5), vascular benefits (Fig. 7), and attenuation of inflammation (Supplementary Fig.7) along with potential important interactions among many of these factors (Fig. 3, Fig. 6, Fig. 7).

Proteomics analysis was performed to elucidate some of the potential molecular mechanisms that could be fundamental to the neuropathological neuroprotection that was observed (Fig. 1). We examined changes in proteins that are potentially important in the neuropathology of HI brain injury. The results of our analyses using next-generation proteomics suggest that treatment with BRT_002 has specific effects in the ipsilateral and contralateral brain hemispheres after exposure to HI in both male and female neonatal rats. Agrin and Zyxin were identified by DEP analysis as common proteins expressed in both the ipsilateral and contralateral brain hemispheres after treatment with BRT_002 compared with vehicle after exposure to HI. Moreover, neurotransmitters and calcium sensors, Syt5, and mitochondrial proteins also appeared to accumulate after treatment with BRT_002. The regulation of co-expressed Agrin, Zyxin and Syt5 in the ipsilateral and contralateral brain hemispheres after treatment with BRT_002 could represent important functions essential for brain tissue repair that could include alterations in the microvasculature, modulation of mitochondrial function, inflammation, and neuronal preservation, contributing to the overall neuroprotection in the animals exposed to HI. The main findings of our study are summarized schematically in Fig. 8.

Treatment with BRT_002 reduced brain injury by reducing infarct volumes in the female and in the total group of neonatal rats after exposure to HI, thereby establishing the neuroprotective efficacy of treatment with BRT_002. Agrin and Zyxin were identified by proteomic analysis, which showed that these proteins were dysregulated when the ipsilateral/contralateral brain hemispheres were compared in the male and female HI-Veh compared with the sham control rats. However, Agrin and Zyxin were also upregulated in both brain hemispheres in the male and female neonatal rats after treatment with BRT_002.

On the other hand, further interpretation of the proteomic profiles showed distinct protein signatures that are involved in the modulation of neurotransmitters and calcium-sensing activity in the groups of rats treated with HI ​+ ​BRT_ 002. These proteins have been associated with neurotransmitter function [64], calcium-sensing [65], regulation of mitochondrial membranes [66], developmental and cellular mitochondria respiration [67], dendrite arborescence, and synaptic transmission or regulation of neuroinflammation [68]. Alterations in the expression patterns of these relevant proteins suggest that they could account for some of the neuroprotective effects provided by BRT_002. Identification of these specific proteins increases our understanding of some of the mechanisms basic to the therapeutic effects of BRT_002.

Moreover, proteomic analysis revealed that Syt5 was significantly higher (p ​< ​0.05) in both the ipsilateral and contralateral hemispheres in the brains of HI-BRT-002 treated total group of male and female neonatal rats compared with the HI-Veh group. The Duolink PLA assay confirmed the co-accumulation of Syt5 with Agrin and Zyxin, and interactions between Syt5 and Vegfa, Vwf, and Ang1, suggesting a potential role for these proteins in the BRT_002 mediated reductions in the infarct volumes in neonatal rats exposed to HI.

Synaptotagmin has an important function in the regulation of membrane traffic [69] Therefore, we performed a study of the relationship between Syt5 and mitochondrial respiration in the BRT-002-treated neonatal rats after exposure to HI. Neurotransmitters and calcium sensors, whose synthesis was augmented by BRT_002, revealed potential interactions between Syt5 and other proteins that were regulated by BRT_002 including Pacs1 and Txn2. These predicted interactions were confirmed by Duolink PLA assays and suggest the probable formation of transient complexes between Syt5 and Txn2. Txn2 is a small protein essential for controlling mitochondrial reactive oxygen species homeostasis and regulating apoptosis and cell viability [70]. Txn2 deficiency impairs mitochondrial redox homeostasis and causes early-onset neurodegeneration with severe cerebellar atrophy, epilepsy, dystonia, optic atrophy, and peripheral neuropathy [70]. Syt5 production was upregulated by treatment with BRT_002 in the ipsilateral brain hemisphere of male and female rats exposed to HI and interactions were observed between Syt5 and Txn2. These findings suggest a potential novel target by which BRT_002 could regulate mitochondrial function thereby facilitating the potential brain repair tissue and neuroprotection.

Networking analysis predicted the interactions between Syt5 and Pacs1 as well as Pacs2. Pacs1 has been described as a regulator of the intrinsic apoptosis pathway and mitochondrial outer membrane permeabilization [71]. Pathogenic variant sites on PACS1 and PACS2 have been associated with neurodevelopmental delay, seizures, behavioral issues, and microcephaly [71]. The key features associated with Pacs2 mutations are intellectual disability, cerebellar dysgenesis and other CNS malformations [72]. Treatment with BRT_002 upregulated Syt5, Pacs1 and Pacs2 in the ipsilateral brain hemisphere of male and female neonatal rats exposed to moderate HI.

LETM1 is a mitochondrial inner membrane protein needed to maintain mitochondrial morphology, cristae structure and regulate mitochondrial ion homeostasis. Although Syt5 and Letm1 proteins have not been shown to interact, we have provided evidence that treatment with BRT_002 affected the regulation of Syt5 and Letm1 in the ipsilateral and contralateral brain hemispheres compared with the vehicle-treated rats after exposure to HI. The results of these findings suggest that BRT_002 promotes the integrity of mitochondria in the brain, protecting the brain by preventing mitochondrial permeabilization, and respiratory deterioration. LETM1 has been associated with optic atrophy 1 (OPA1), and its dysregulation in HI could destabilize L-OPA1 and cause mitochondrial fragmentation, disturb the mitochondrial ultrastructure, and increase apoptotic sensitivity [73]. Moreover, the results of our study can be interpreted to suggest that by stimulating these protective mechanisms, BRT_002 attenuates HI-related brain injury.

We also provide evidence that BRT_002 provides neuroprotection by increasing mitochondrial respiration and producing metabolic changes in mouse neurons exposed *in vitro* to oxygen-glucose deprivation. BRT_002 mediated neuroprotection was also suggested *in vivo* because exposure to HI was associated with decreases in the expression of the dendrite marker (Map2) and mature neuronal marker (Neun), whereas treatment with BRT_002 reduced neuronal death, as suggested by increases in the glutamatergic (Slc17a7) and GABAergic markers in the ipsilateral brain hemispheres of male and female neonatal rats after treatment with BRT_002. These findings suggest that Syt5 and proteins associated with mitochondrial dynamics collectively modulate oxidative stress to improve neuronal survival.

Agrin is a heparan sulfate proteoglycan widely expressed at intraneuronal synapses and required for the establishment of a tight BBB. This suggests that Agrin could potentially be important in the attenuation of brain lesions after exposure to HI. The importance of Agrin in brain repair tissue has previously been reported by Ye Wang and colleagues [74]. They demonstrated that intrathecal administration of Agrin resulted in its spatiotemporal brain distribution, and inhibited BBB disruption by reducing the loss of tight-junctional proteins after exposure of adult mice to ischemia/reperfusion. These changes were associated with reductions in infarct volume, decreases in apoptotic neurons, and improvements in neurological function. The findings of this study are consistent with previous observations suggesting that Agrin contributes to the barrier properties of endothelial cells and stabilizes tight connections in the BBB [75,76]. Agrin has also been reported to facilitate coordinated therapeutic processes, which improve cardiac repair in pigs [77]. Consequently, the upregulation of Agrin could have contributed to the neuroprotection observed after treatment with BRT_002 of neonatal rats exposed to HI-related brain injury.

Zyxin is involved in regulating the secretion of endothelial VWF, which participates in the repair of brain lesions after HI-related insults [49]. The PLA study demonstrated in situ interactions between Zyxin and Agrin, suggesting their coordinated roles in brain tissue repair. Their molecular interactions and their connections with Vegfa reinforce their participation in the brain tissue repair processes. VWF is secreted locally as a result of cellular activation for brain repair [78,79] and VEGF regulates the exocytosis of VWF, after CNS microvasculature injury [80]. Ipsilateral expression of Vwf was markedly increased in the brains of the HI-BRT_002 treated compared with the HI-Veh male and female neonatal rats after injury. RT‒qPCR also revealed significant increases in the ipsilateral expression of Vegfa in the HI-BRT_002 compared with the HI-Veh treated females, males, and the total group of female plus male neonatal rats. In this regard, modulation of Agrin and Zyxin expression in the brains of male and female neonatal rats exposed to HI could have implications for the therapeutic role of neuroprotection.

Numerous studies have shown that the modulation of mitochondrial function attenuates neuroinflammation [81] and provides neuroprotection [82,83]. The increases in Agrin, Zyxin and Syt5 in the ipsilateral brain hemispheres of male and female neonatal rats were associated with decreases in astrocytic and microglial markers in the BRT_002-treated compared with the HI-Veh group. Although previous reports have shown increases in BBB permeability [84] and neurodegeneration after exposure to HI in neonatal rats, the association between markers of inflammation and the potential modulation of Syt5, Agrin and Zyxin require further investigation.

There are several opportunities for future research and limitations to our study. Although we have shown the novel BRT_ 002 purine derivative has important neuroprotective effects, increases the signatures of multiple molecular agents that could account for its neuroprotective efficacy, and improves short term locomotion in neonatal rats, future studies with this novel purine derivative are needed to determine if other dosages, and/or different dosing regimen can further augment its protective effects. Furthermore, additional studies could increase our understanding of the other mechanisms underlying the neuroprotection provided by BRT_ 002. Even though BRT_ 002 appears to provide short term benefits for early locomotion, additional studies are required for more long-term neurobehavioral and cognitive outcomes beyond the neonatal period. These studies would require long-term survival into adulthood after exposure of the neonates to HI with and without treatment with BRT_ 002.

In summary, our study provides *in vivo* evidence that our newly developed novel substituted purine derivative BRT_002 attenuated brain injury after exposure of neonatal rats to an episode of HI. The mechanisms by which BRT_002 mediated its beneficial therapeutic effects were elucidated using a multidisciplinary approach to examine changes after HI at the protein and cellular levels, studying vascular effects, mitochondrial function, and neuroinflammation to determine some of the mechanisms that form the basis for the neuroprotective effects provided by BRT_002. Our findings provide encouraging preclinical data that could potentially serve as a basis for future clinical trials of this novel purine derivative as a potential pharmacological agent to treat HIE in newborns.

## Consent for publication

Not applicable.

## Availability of data and materials

Data will be made available on request. Mass spectrometry proteomics data corresponding to the 60 nanoLC‒MS/MS runs were deposited at the ProteomeXchange Consortium via the PRIDE partner repository (https://www.ebi.ac.uk/pride/) under dataset identifiers: "Project accession PXD050059"

## Authors contributions

**AM** was responsible for project conceptualization, administration, NIH and ANR funding acquisition, data analysis and writing of the original manuscript. **CD** conducted *in vivo* experiments, analyzed their data and prepared their figures and the writing of the manuscript. **AB**: contributed to the proteomic data analysis, generation of the figures, and the writing and reviewing of the original manuscript. **KSL** contributed to the Duolink proximity ligation assay. **NO** and **HG** were involved in drug synthesis. **BK, ALM** and **XC** contributed *in vivo* experiments, analyzed their data. **NC, ALM** contributed to the Q-PCR experiments. **GV** and **HH** contributed to the mitochondrial respiration experiments and their data analysis. **AP** and **LNG** contributed to the bioanalysis and pharmacokinetics analysis of purine derivatives in brain, and plasma samples. **AT** contributed to the behavioral tests, analysis and preparation of corresponding figures. **JA** contributed to the generation of proteomic data. **RH** and **RAH** contributed to the bioinformatics data analysis. **PG** contributed to the generation of slices for duolink experiments and in the discussion for the writing of the manuscript. **BSS** received the NIH funding for the project and was responsible for the conceptualization of the project, data analysis, and writing of the manuscript. All the authors have reviewed the manuscript.

## Funding

The current study was supported by an 10.13039/100000002NIH grant (1R01NS117428-01A1), by ANR (ANR-21-CE17-0019-01), as well as by ​Fondation Grace de Monaco, ANR-23-IAHU-0010 (France 2030), and an additional grant from “Investissement d'Avenir -ANR-11-INBS-0011-” NeurATRIS. The authors assume all responsibility for the study and assert that the contents herein do not represent the National Institutes of Health's official views.

## Declaration of competing interest

The authors declare the following financial interests/personal relationships which may be considered as potential competing interests: MABONDZO reports financial support was provided by Paris-Saclay University. MABONDZO reports was provided by National Institute of Health. MABONDZO reports financial support, administrative support, article publishing charges, equipment, drugs, or supplies, statistical analysis, travel, and writing assistance were provided by National Institutes of Health. Reports a relationship with that includes:. Has patent pending to. If there are other authors, they declare that they have no known competing financial interests or personal relationships that could have appeared to influence the work reported in this paper.
