# Supplementary material for: Novel purine derivatives mitigate hypoxia ischemia related brain injury through agrin, zyxin and synaptotagmin proteins
Source: Neurotherapeutics. 2025 Jun 17;22(5):e00621. doi: 10.1016/j.neurot.2025.e00621 (PMC12491796; doi:10.1016/j.neurot.2025.e00621)
Supplement: Multimedia component 1 [file mmc1.docx]

#### Supplementary material

**Supplementary Figure 1: Synthesis of BRT_001 and its valyl ester, BRT_002.**

Trisubstituted purine derivative BRT_001 (compound **4**) was prepared from 2,6-dichloropurine in three steps. N-Ethylbenzylamine was heated with 2,6-dichloropurine and triethylamine in *iso-*propanol to obtain **2**. In the second step, selective alkylation of **2** occurred at position 9 of the purine to yield derivative **3**, which was heated with R-aminobutanol without added solvent to obtain **4**. Boc-valine was activated by a mixture of 1-hydroxybenzotriazole (HOBt) and dicyclohexylcarbodiimide (DCC) in the next step. The active ester was reacted with **4** to yield **5**. Deprotection of **5** with HCl in Et_2_O afforded BRT_002, which was isolated as a hydrochloride.

Reagents and conditions. (a) N-Ethylbenzylamine, NEt_3_, isoPrOH, 3 h, 85 °C; (b) K_2_CO_3_, 2-bromopropane, DMSO, 24 h, 20 °C; (c) (R)-2-aminobutan-1-ol, 160 °C, 6 h; (d) Boc-valine, DCC, HOBt, AcOEt, THF, 3 h ; (e) HCl in Et_2_O, 20 °C.

**N-Benzyl-2-chloro-N-ethyl-9H-purin-6-amine *(2)***

2,6-Dichloropurine, **1** (9.6 g, 41.5 mmol), was added to a solution of N-ethylbenzylamine (8.4 mL, 62.2 mmol) and NEt_3_ (8.4 mL, 62.2 mmol) in *i*-PrOH (150 mL) while stirring. The mixture was heated for 3 h at 85 °C. After cooling, the crystallized solid was filtered and washed twice (10 mL, 10 °C). After drying, compound **2** was found to be pure in a vacuum by TLC (cyclohexane-CH_2_Cl_2_, 6:4; Rf 0.8 compared to **1** Rf 0.25). Yield 66%. ^1^H-NMR (400 MHz, DMSO-*d*_6_, δ ppm): 1.17 (brs, 3H), 3.58 and 4.16 (2 brs, 2H), 4.93 and 4.58 (2 brs, 2H), 7.29 (brs, 5H), 8.15 (s, 1H).

**N-Benzyl-2-chloro-N-ethyl-9-isopropyl-purin-6-amine *(3)***

A solution of **2** (4.98, 49.7 mmol) in DMSO (75 mL) was cooled at 15 °C. K_2_CO_3_ (27.5 g, 19.9 mmol) was added under stirring. After 5 min at 15 °C, 2-bromopropane (23.2 mL, 24.8 mmol) was introduced slowly. The mixture was stirred and maintained at 20 °C overnight. The solution was cooled to 10 °C, and 200 mL of cold water was added. The mixture was extracted with AcOEt (3x20 mL). The organic layer was washed with brine (2x50 mL) and H_2_O (25 mL). The solution was dried over Na_2_SO_4_ and concentrated in vacuo to yield **3** as a white solid, which was triturated with 3 mL of AcOEt and isolated by filtration on a Büchner funnel. Yield 82%. ^1^H-NMR (400 MHz, DMSO-*d*_6_, δ ppm): 1.18 (brs, 3H), 1.51 (d, *J* =6.5 Hz, 6H), 3.58 and 4.15 (2 brs, 2H), 4.70 (hept, *J* =6.5 Hz, 1H), 4.91 and 5.55 (2 brs, 2H), 7.32 (m, 5H), 8.30 (s, 1H).

**(2R)-2-[[6-[Benzyl(ethyl)amino]-9-isopropyl-purin-2-yl]amino]butan-1-ol (*4*)**

A mixture of **3** (5.72, 17.3 mmol) and (2R)-aminobutanol (16.3 mL, 17.3 mmol) was heated at 160 °C for 6 h. The reaction was monitored by TLC. After completion, the mixture was cooled and partitioned with a mixture of H_2_O (20 mL) and AcOEt (3x30 mL). The organic layer was washed with brine (2x20 mL) and H_2_O (20 mL). After drying over Na_2_SO_4_, the solution was evaporated under a vacuum. The solid was triturated with AcOEt (3 mL). The solid was filtered with a Büchner filter and dried under a vacuum. Yield 75%. ^1^H-NMR (400 MHz, DMSO-*d*_6_, δ ppm): 0.78 (t, 3H, *J = 6.5 Hz*), 0.78 (brs, 3H), 1.06 (t, 3H, *J = 6.5 Hz*), 1.39 (d, 6H, *J = 6 Hz*), 1.53 (m, 2H), 3.41 (m, 1H), 3.71 (m, 1H), 5.15 (brs, 2H), 5.78 (brd 1H), 7.22 (brs, 5H), 7.74 (s, 1H).

**[(2R)-2-[[6-[Benzyl(ethyl)amino]-9-isopropyl-purin-2-yl]amino]butyl]**

**(2S)-2-(*tert*-butoxycarbonylamino)-3-methyl-butanoate *(5)***

Dicyclohexylcarbodiimide (2.44 g, 12 mmol) was added at 5 °C to a solution of *tert-*butyloxycarbonylvaline, Boc-valine (2.60 g, 12 mmol), and 1-hydroxybenzotriazole (HOBt) (1.62 g, 12 mmol) in 60 mL of AcOEt. The cooling bath was removed after 5 min, and stirring at 20 °C continued for 3 h. The suspension was filtered through a Büchner filter, and the precipitate (DCU) was washed with AcOEt (2x5 mL). The combined filtrates were added to a mixture of compound **4** (3.8 g, 10 mmol) and NEt_3_ (6 mL, 20 mmol) in THF (50 mL). Stirring was continued at 20 °C for 2 days. The mixture was transferred to a separatory funnel and washed with 25 mL of 1 M Na_2_CO_3_, followed by 50 mL of brine and 50 mL of H_2_O. The organic layer was dried over Na_2_SO_4_ and concentrated under a vacuum below 40 °C. The ester crystallized and then was triturated with 5 mL of AcOEt and isolated by filtration on a Büchner filter. Yield: 65%. ^1^H-NMR (400 MHz, DMSO-*d*_6_, δ ppm): 0.75 (3H, t, *J* =6.5 Hz), 1.12 (m, 3H), 1.38 (d, 6H), 1.54 (m, 17H), 1.95 (m, 1H), 3.65 (m, 1H), 4.05 (m, 4H), 4.55 (1H, Hept, *J* = 6.5 Hz), 5.05 and 5.45 (2 brs, 2H), 6.25 (brs, 1H), 7.25 (m, 5H), 7.80 (s, 1H).

**[(2R)-2-[[6-[Benzyl(ethyl)amino]-9-isopropyl-purin-2-yl]amino]butyl](2S)-2-amino-3-methyl-butanoate hydrochloride or the L-valyl ester of (2R)-2-[[6-[benzyl(ethyl)amino]-9-isopropyl-purin-2-yl]amino]butan-1-ol hydrochloride (*BRT_002*)**

Ester **5** (1.45 g, 2.5 mmol**)** was dissolved in 50 mL of anhydrous Et_2_O. A 2 M HCl solution in Et_2_O (20 mL) was added and stirred at 20 °C for 24 h. The solvent was decanted, and the solid was triturated with Et_2_O (3x20 mL). The precipitate was then dried under a vacuum (P_2_O_5_) for 48 h to yield **BRT_002**. Yield: 43%. ^1^H-NMR (400 MHz, DMSO-*d*_6_, δ ppm): 0.75 (m, 3H), 1.15 (m, 3H), 1.45 (m, 6H), 1.64 (m, 8H), 2.35 (m, 1H), 3.75 (m, 1H), 4.12 (m, 4H), 4.55 (m, 1H), 4.95 and 5.45 (2 brs, 2H), 6.25 (brs, 1H), 7.25 (m, 5H), 8.20 (brs, 1H), 8.45 (m, 3H).


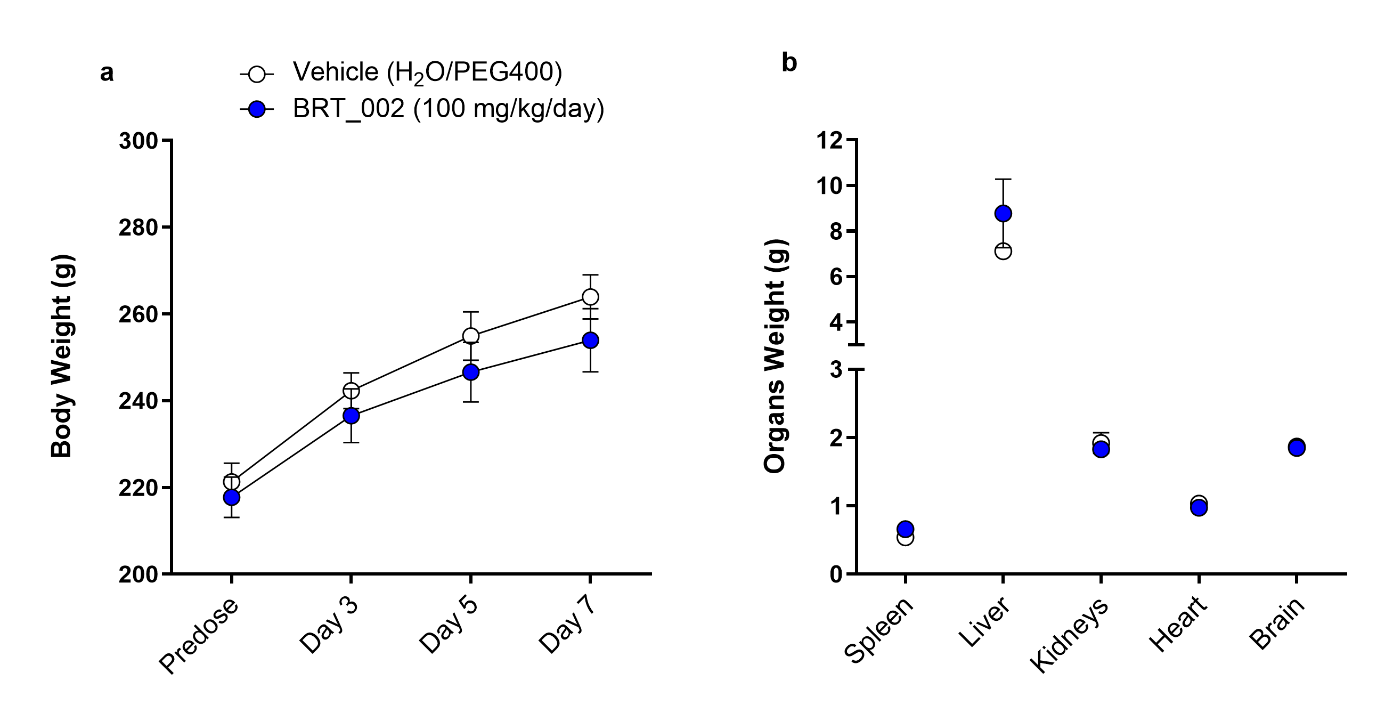


**Supplementary Figure 2: Safety of BRT-002.** Changes in **(a)** body weight over 7 days of daily oral administration of BRT_002 (100 mg/kg) or vehicle (H_2_O/PEG400) (n=5 animals/group), **(b)** organs weights on day 7 in adult male Wistar rats (n=2-3 animals/group). Statistical analysis are by Two-way ANOVA (factors were time and treatment). Statically significant differences in body weight over the study days or organ weights between the Vehicle and BRT_002 treated groups were not detected.


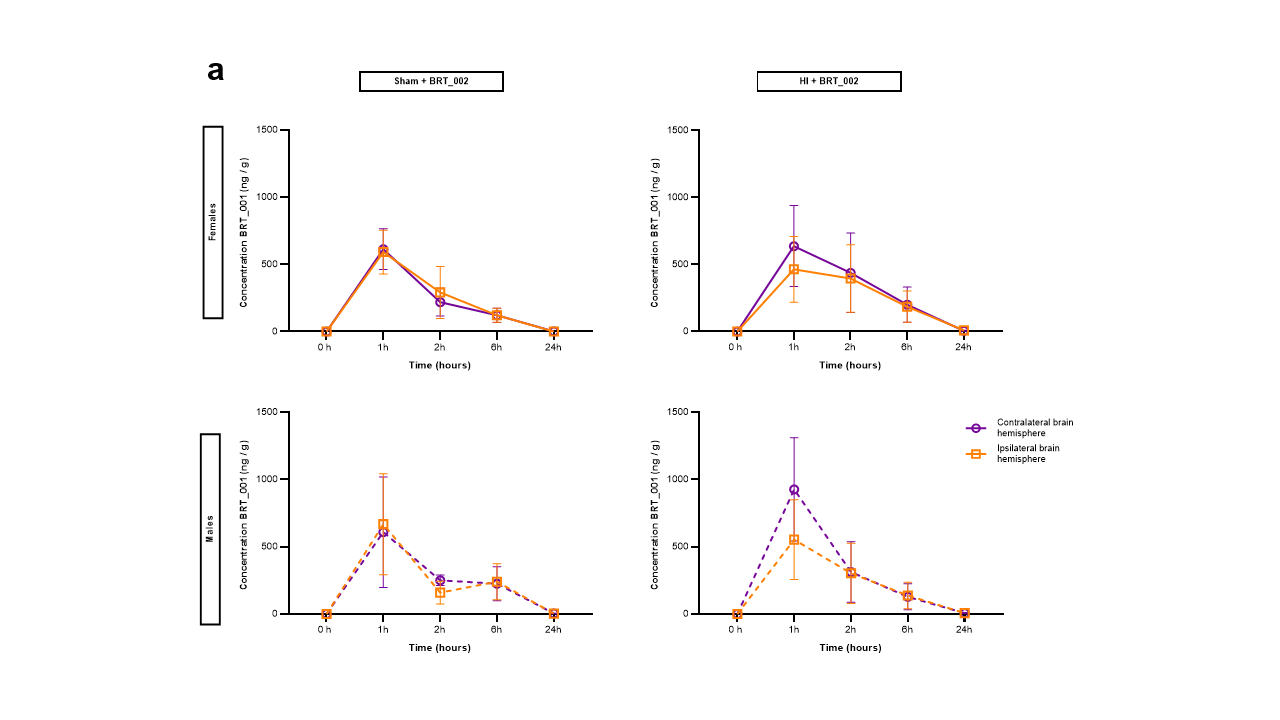


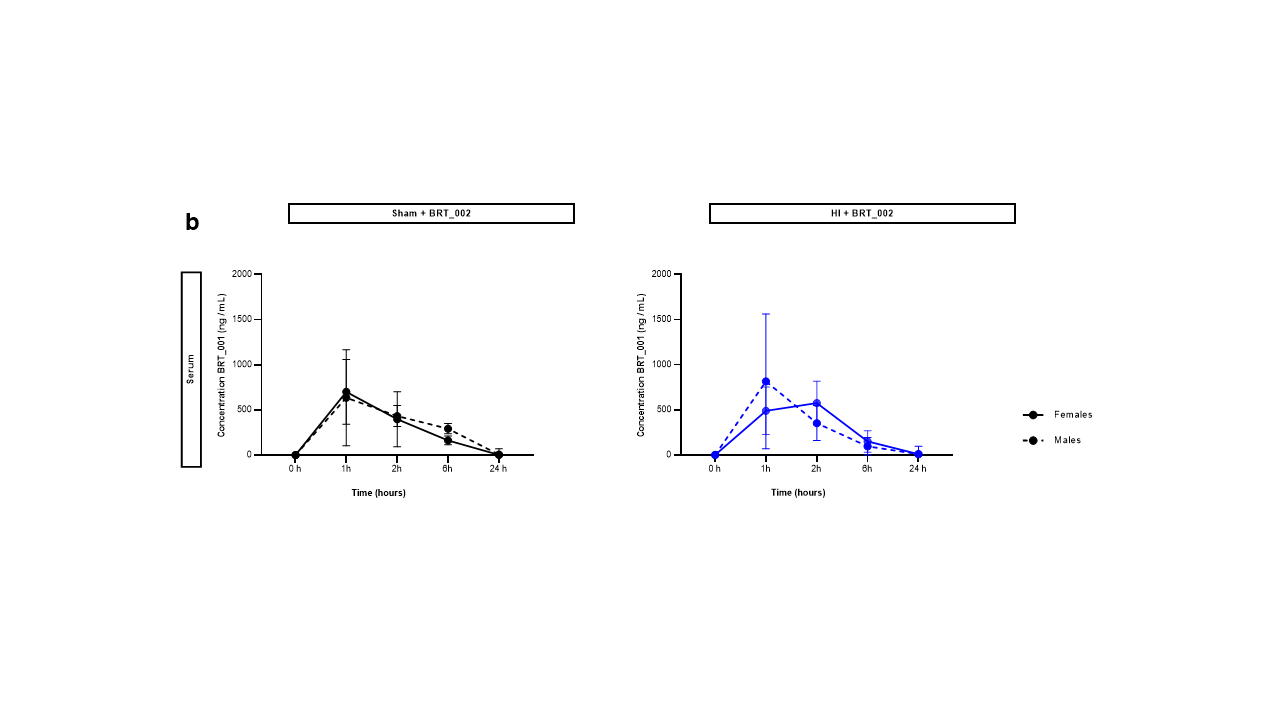


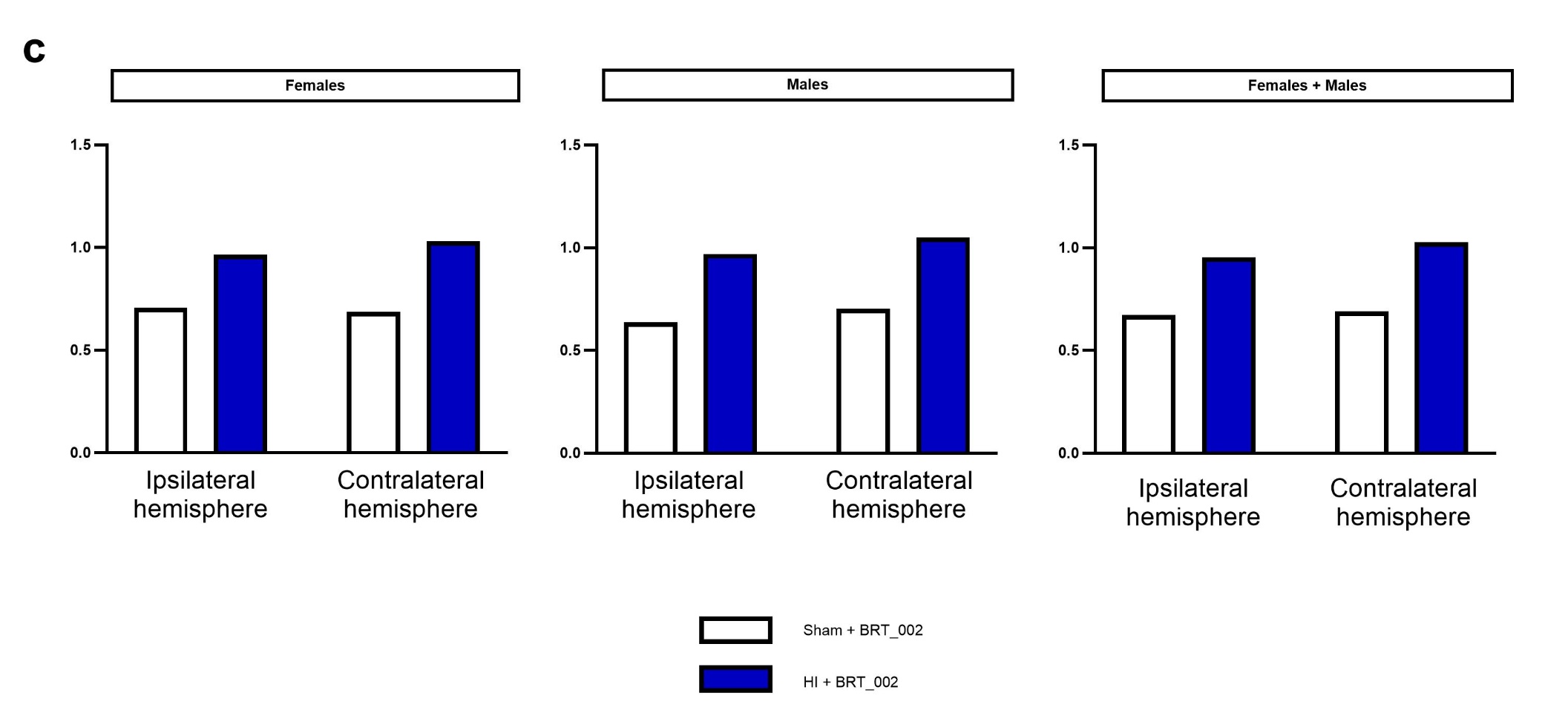


**Supplementary Figure 3: Pharmacokinetic profiles of BRT_001. (a)** Concentrations of BRT_001 in the ipsilateral and contralateral hemispheres of the brain after a single IP administration of 30 mg/kg BRT_002 in male and female neonatal rats in the sham and HI groups. Statistics are: Two-way ANOVA (factors were hemispheres: ipsilateral/contralateral and time): Statistical differences were not identified. (**b)** Concentrations of BRT_001 in serum; Two-way ANOVA (factors were sex and time): Statistical differences were not identified. Data are shown as mean ± s.e.m. n =6 per group. Each point represents 6 animals. **(c**) Partition coefficient (Kp= AUC brain/AUC serum).

**
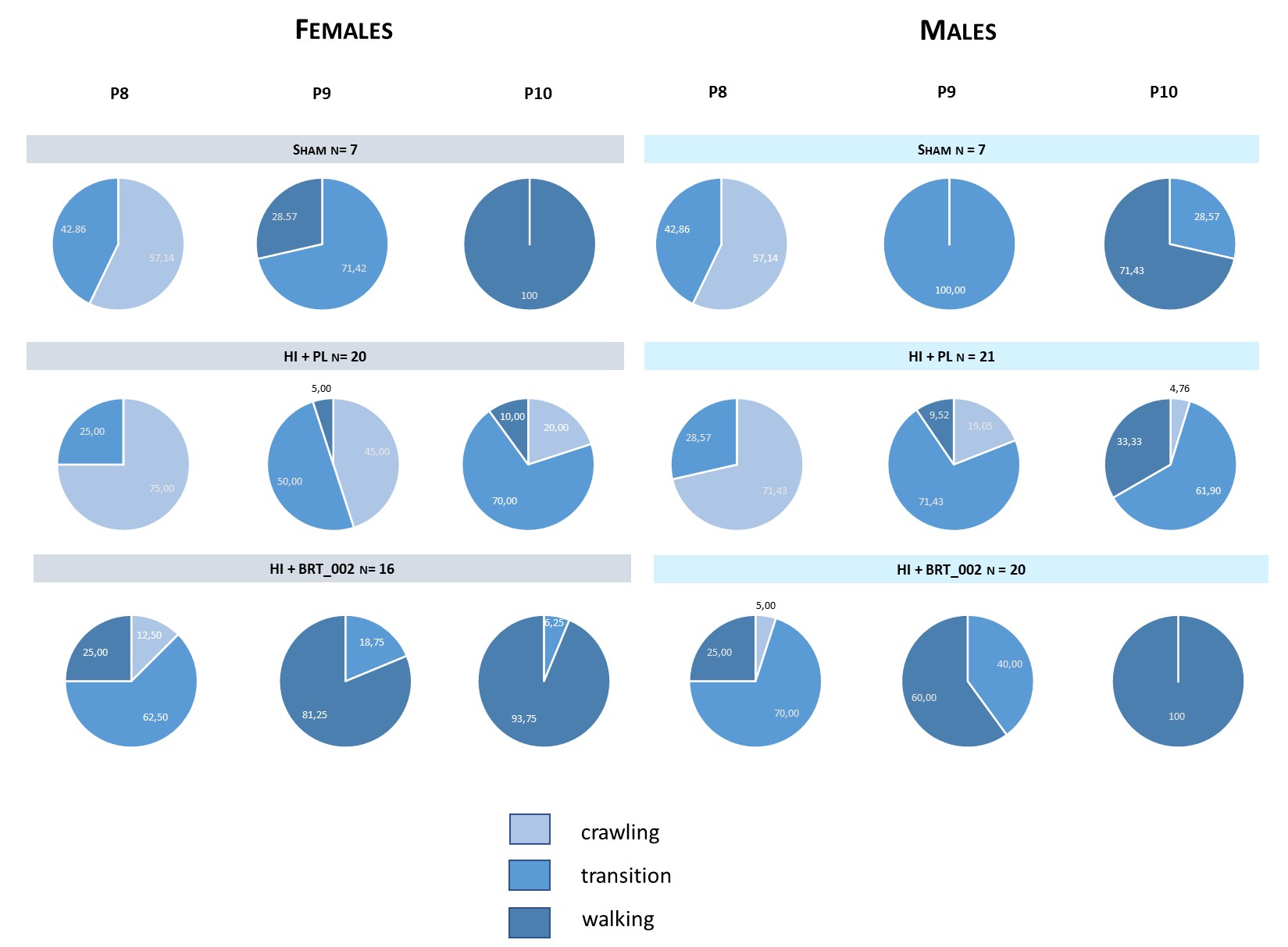
**

**Supplementary Figure 4: The effects of BRT_002 on behavioral outcomes in neonatal rats exposed to HI related brain injury.** The ambulation test (P8-P10) in females and males with the crawling in light blue, the transition in blue and the beginning of walk in dark blue.. In females the animal number used was: Sham n = 7, HI+PL n=20, HI+BRT_002 n=16 and in males : Sham n = 7, HI+PL n=21, HI+BRT-002 n=20.

**
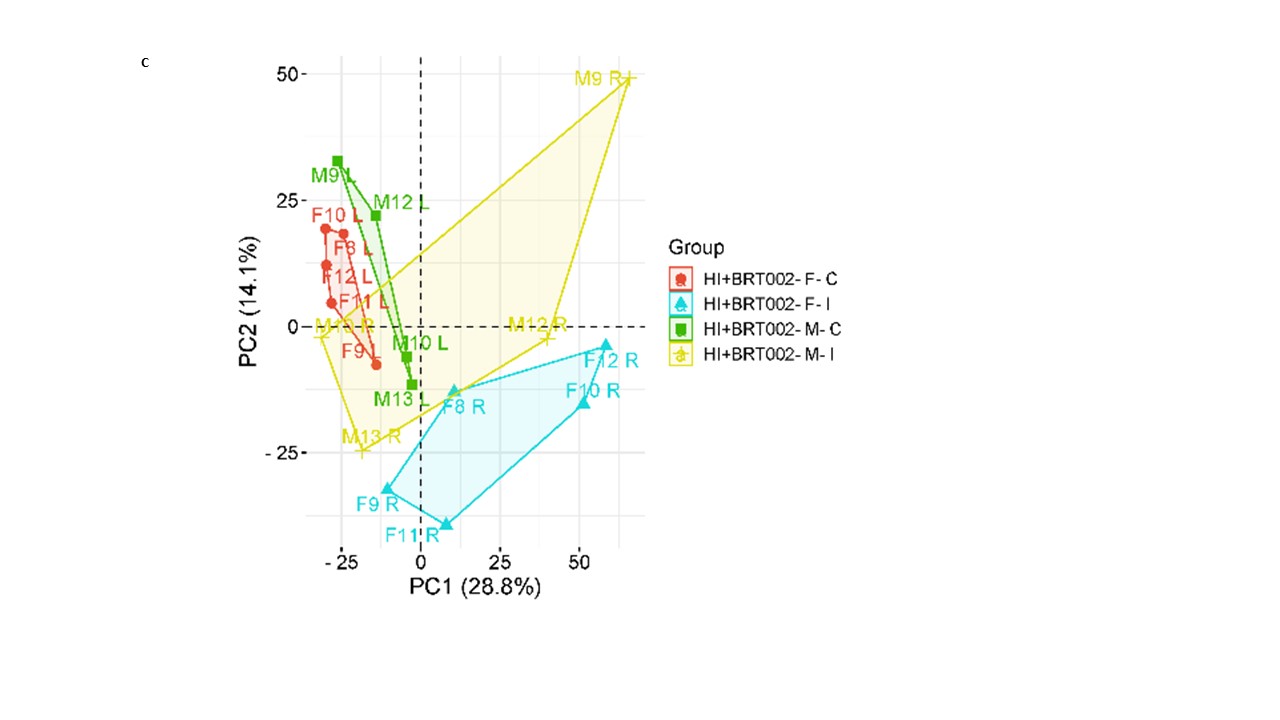
**
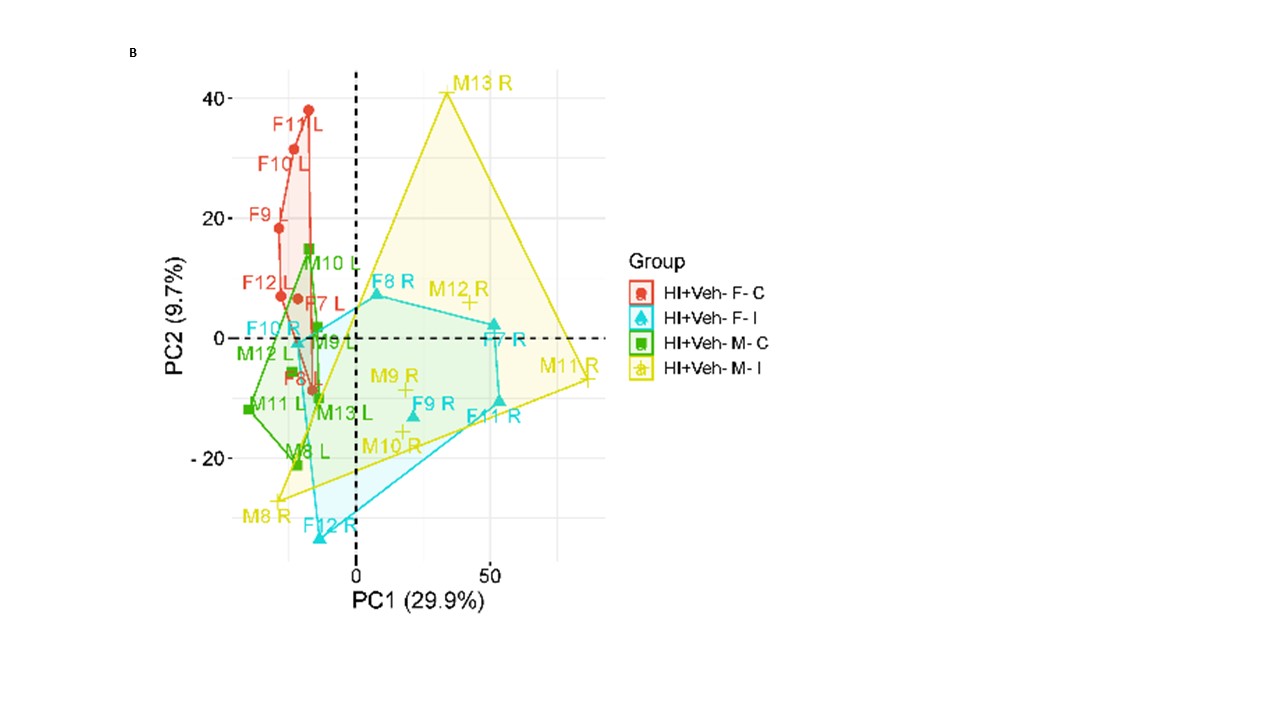

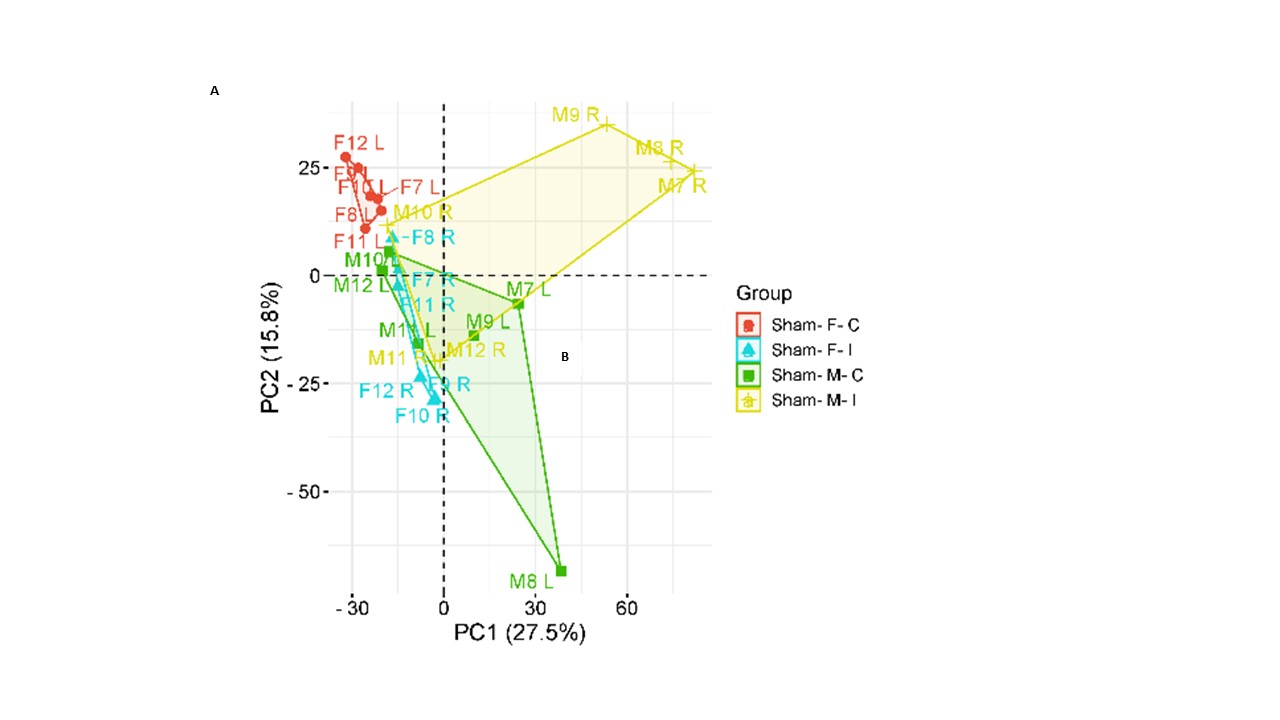


**Supplementary Figure 5: Quality control analysis of proteomics data: Principal component analysis (PCA) revealed distinct clustering of the a) sham groups, b) HI+Vehicle groups and c) HI+BRT_002 groups of neonatal rats.**

Sham, HI+Vehicle and HI+BRT_002 neonatal rats, four clusters corresponding to female contralateral hemisphere samples (cluster 1, red), female ipsilateral hemisphere samples (cluster 2, blue), male contralateral hemisphere samples (cluster 3, green) and male ipsilateral hemisphere samples (cluster 4, yellow) were identified in each group of neonatal rats. F: female, M: male, C: contralateral, I: ipsilateral.

**
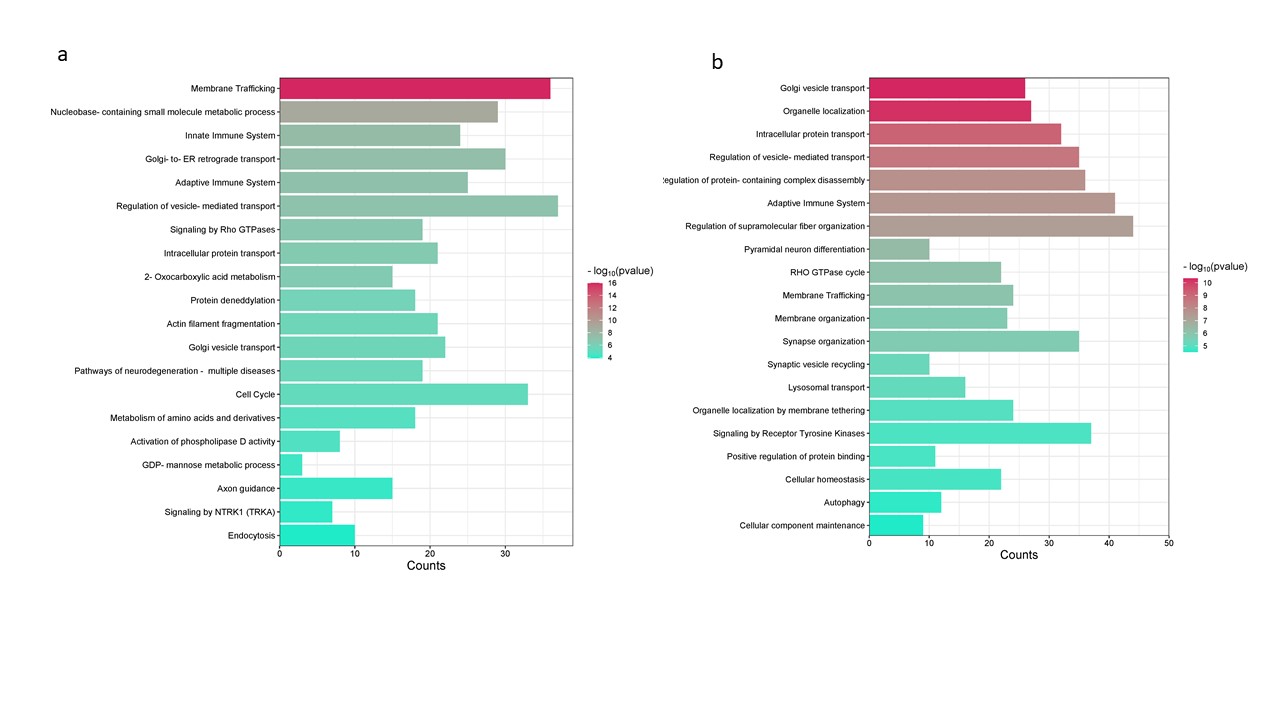
**

**
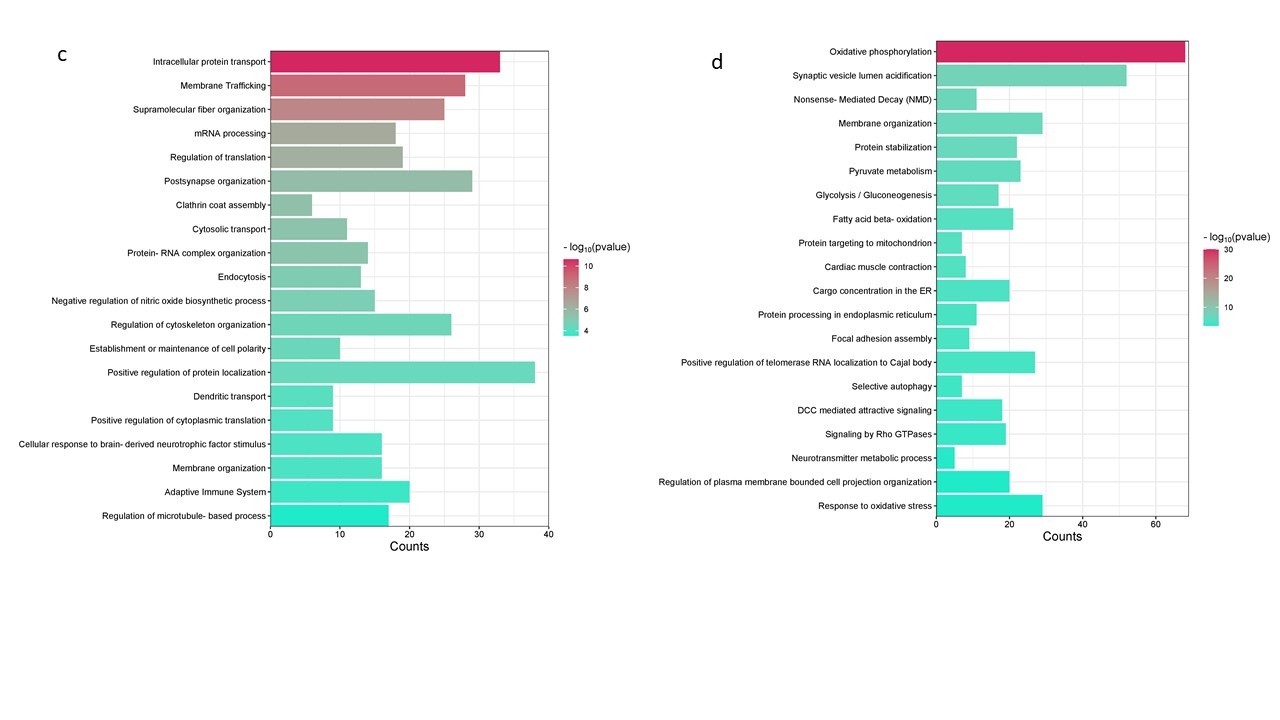
**

**
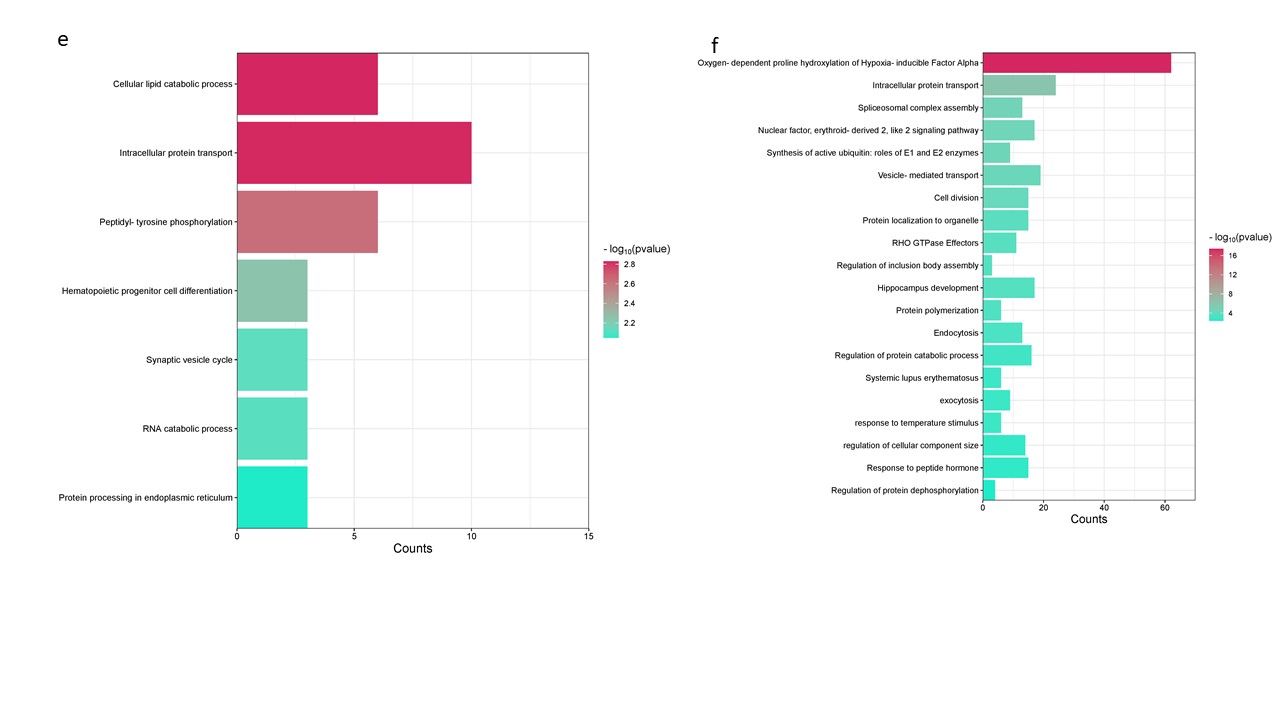
**

**
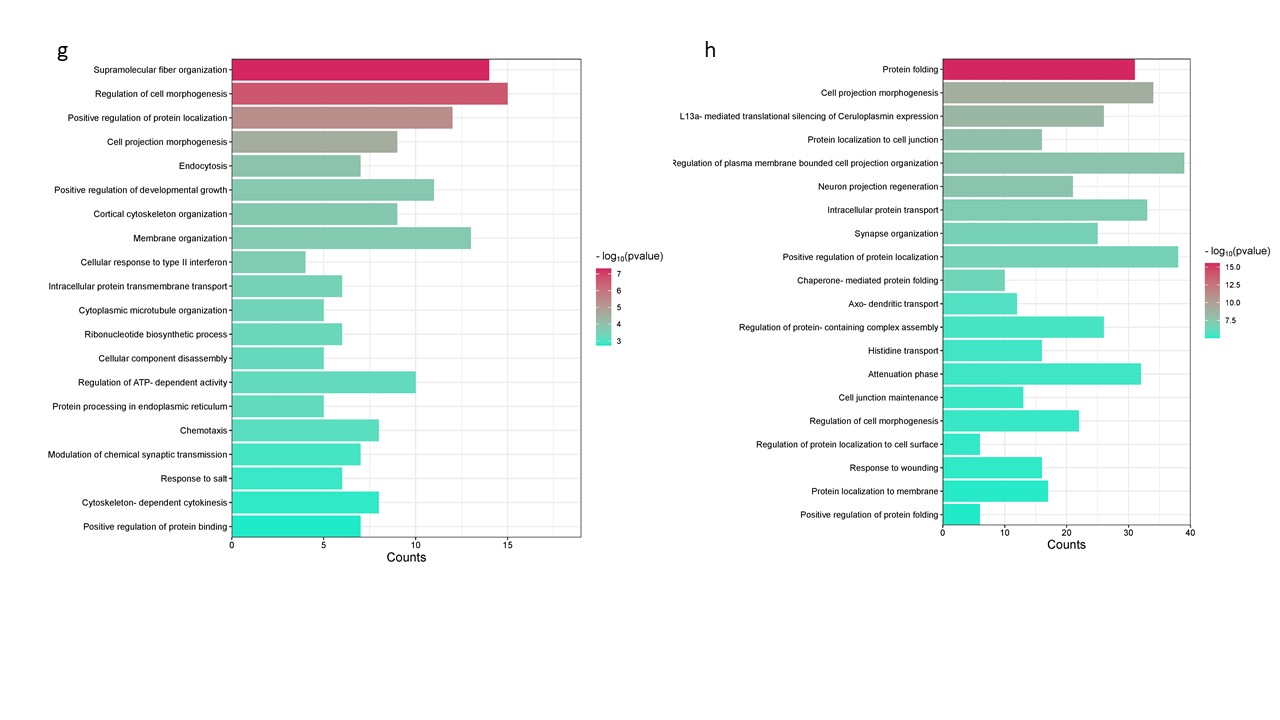
**

**
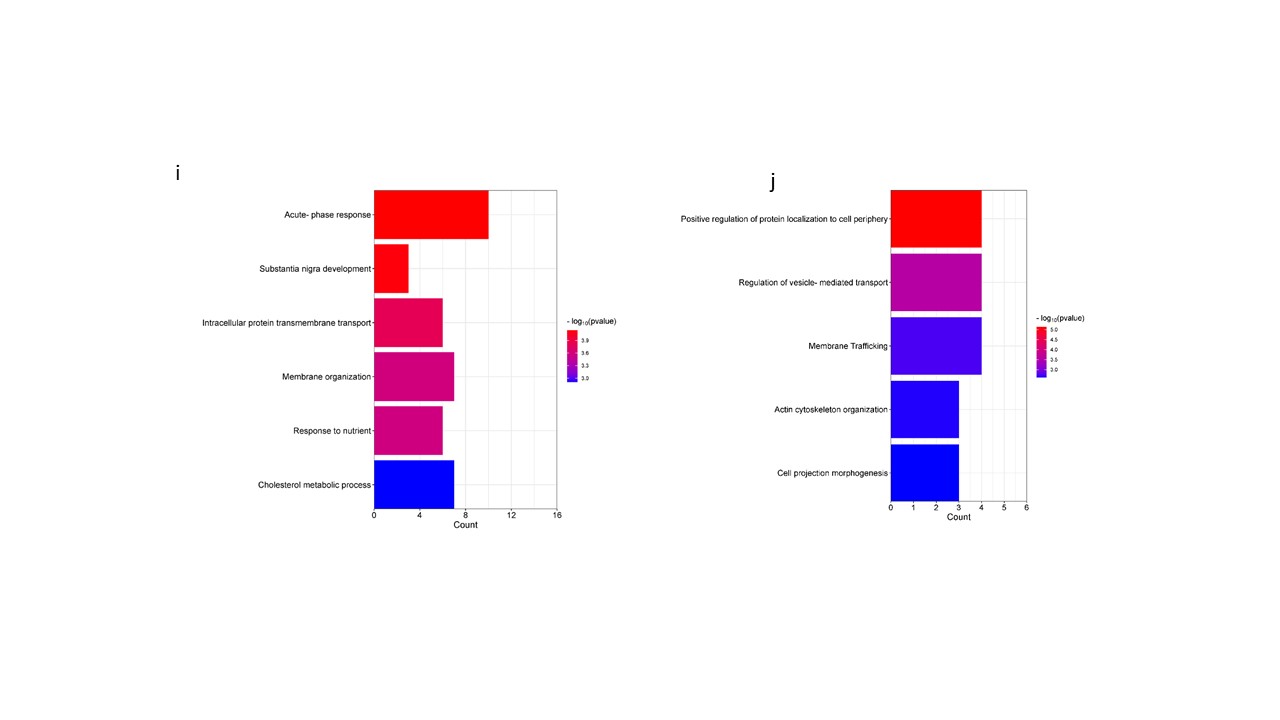
**

**Supplementary Figure 6: Functional enrichment analysis of the** **a**) up and **b**) down expressed proteins in HI+BRT_002 compared to HI+Vehicle in the contralateral hemisphere of female neonatal rats. **c)** up- and **d**) downregulated proteins in the ipsilateral hemisphere of female neonatal rats in the HI+BRT_002 group compared to the HI+Vehicle group. **e**) up- and **f**) downregulated proteins in the contralateral hemisphere of male neonatal rats in the HI+BRT_002 group compared to the HI+Vehicle group. **g**) up- and h) downregulated proteins in the ipsilateral hemisphere of male neonatal rats in the HI+BRT_002 group compared to the HI+Vehicle group. **i**) in the contralateral hemisphere and **j**) ipsilateral hemisphere of a mixture of male and female rats in the HI+BRT_002 group compared to the HI+Vehicle group.


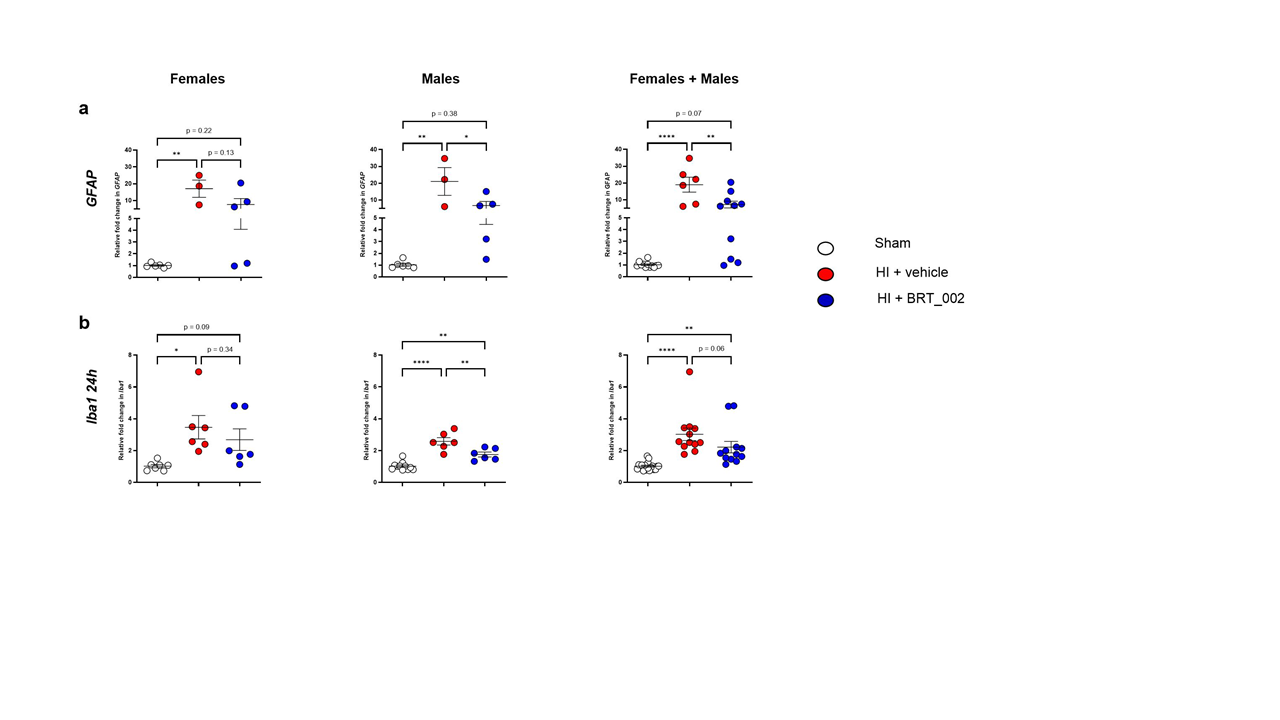


**Supplementary Figure 7: BRT_002 attenuates markers of neuroinflammation in neonatal rats after exposure to moderate HI in the ipsilateral brain hemisphere of female and male neonatal rats. (a)** mRNA expression of *GFAP* 72 h after HI injury. (**b)** mRNA expression of *Iba1* 24 h after HI injury*.* Sham is designated by open circles, HI Vehicle is designated by closed red circles, and HI+BRT_002 (30 mg/kg) is designated by closed blue circles. Data for females (sham, n=6; HI + Vehicle, n= 3 and HI + BRT_002, n=5), males (sham, n=6; HI + Vehicle, n= 3 and HI + BRT_002, n=5) and males + females (sham, n=12; HI + Vehicle, n= 6 and HI + BRT_002, n=10) were compared to those in the control and vehicle groups. *P < 0.05; **P < 0.01; ***P < 0.001; **** P < 0.0001, one-way ANOVA, Tukey’s multiple comparison test**.**

**Supplementary Table 1: Drug safety validation.** (a) Hematology parameters WBC: White Blood Cells; NEUT: Neutrophil; EOSI: Eosinophil; BASO: Basophil; LYMP: Lymphocyte; MONO: Monocyte; LUC: Large Unstained Cell; RBC: Red Blood Cell; HGB: Hemoglobin; HCT: Hematocrit; RET: Reticulocytes; RDW: Red Distribution Width; MCV: Mean Corpuscular Volume; MCH: Mean Corpuscular Haemoglobin; MCHC: Mean Corpuscular Haemoglobin Concentration; THR: Thrombocyte Count; MPV: Mean Platelet Volume; APTT: Activated Partial Thromboplastin Time; PT: Prothrombin Time. P>0.05, *: P<0.05, **: P<0.01, compared to the control group. (b) Clinical chemistry parameters; ALT: Alanine Aminotransferase; AST: Aspartate Aminotransferase; ALP: Alkaline phosphatase; TBIL: Total Bilirubin; PROT: Total Protein; ALB: Albumin; CREA: Creatinine; UREA: Urea nitrogen; GLU: Glucose; CHOL: Total Cholesterol; TRIG: Triglyceride; Ca: Calcium; Cl: Chloride; K: Potassium; Na: Sodium; GLOB: Globulin; AG: Ratio Albumin/Globulin; NS: P>0.05, *: P<0.05, **: P<0.01, compared to the control group.

| **a** |  |  |  |  |  |  |  |  |  |  |  |  |  |  |  |  |  |  |  |  |
| --- | --- | --- | --- | --- | --- | --- | --- | --- | --- | --- | --- | --- | --- | --- | --- | --- | --- | --- | --- | --- |
| **Treatment** |  | **WBC** | **NEUT** | **EOSI** | **BASO** | **LYMP** | **MONO** | **LUC** | **RBC** | **HGB** | **HCT** | **RET** | **RDW** | **MCV** | **MCH** | **MCHC** | **THR** | **MPV** | **APTT** | **PT** |
| **Vehicle** | Mean | 4.7 | 1.12 | 0.06 | 0.01 | 3.5 | 0.1 | 0.02 | 6.8 | 13.3 | 39 | 292 | 12.5 | 58 | 19.6 | 34 | 725 | 7.3 | 21.4 | 20.2 |
|  | SEM | 1.5 | 0.07 | 0.02 | 0 | 1 | 0.02 | 0.01 | 0.5 | 1.1 | 3 | 21 | 0.6 | 1 | 0.3 | 0 | 54 | 0.2 | 2.8 | 1.1 |
|  | N | 5 | 5 | 5 | 5 | 5 | 5 | 5 | 5 | 5 | 5 | 5 | 5 | 5 | 5 | 5 | 5 | 5 | 5 | 5 |
| **BRT_002 (100 mg/kg/day)** | Mean | 5.7 | 1.09 | 0.05 | 0.01 | 4.4 | 0.11 | 0.03 | 7.3 | 14.1 | 41 | 297 | 14.7 | 57 | 19.3 | 34 | 550 | 7.5 | 24.6 | 21.6 |
|  | SEM | 0.9 | 0.28 | 0.01 | 0.01 | 0.9 | 0.02 | 0.01 | 0.3 | 0.8 | 2 | 28 | 2.6 | 1 | 0.6 | 1 | 123 | 0.5 | 4.5 | 2.8 |
|  | % | 5 | 5 | 5 | 5 | 5 | 5 | 5 | 5 | 5 | 5 | 5 | 5 | 5 | 5 | 5 | 5 | 5 | 4 | 5 |
|  | N | 21 | -3 | -17 | 0 | 26 | 10 | 50 | 7 | 6 | 5 | 2 | 18 | -2 | -2 | 0 | -24 | 3 | 15 | 7 |
|  | P | NS | NS | NS | NS | NS | NS | NS | NS | NS | NS | NS | NS | NS | NS | NS | NS | NS | NS | NS |
|  | Threshold | 2.8 | 0.81 | 0.06 | 0.02 | 2.9 | 0.08 | 0.03 | 1.5 | 3.5 | 9 | 86 | 5.5 | 3 | 1.4 | 2 | 303 | 1.1 | 10.1 | 6.2 |
|  |  |  |  |  |  |  |  |  |  |  |  |  |  |  |  |  |  |  |  |  |
|  |  |  |  |  |  |  |  |  |  |  |  |  |  |  |  |  |  |  |  |  |
|  |  |  |  |  |  |  |  |  |  |  |  |  |  |  |  |  |  |  |  |  |
|  |  |  |  |  |  |  |  |  |  |  |  |  |  |  |  |  |  |  |  |  |
|  |  |  |  |  |  |  |  |  |  |  |  |  |  |  |  |  |  |  |  |  |
|  |  |  |  |  |  |  |  |  |  |  |  |  |  |  |  |  |  |  |  |  |
|  |  |  |  |  |  |  |  |  |  |  |  |  |  |  |  |  |  |  |  |  |
| **b** |  |  |  |  |  |  |  |  |  |  |  |  |  |  |  |  |  |  |  |  |
| **Treatment** |  | **ALT** | **AST** | **ALP** | **TBIL** | **PROT** | **ALB** | **CREA** | **UREA** | **GLU** | **CHOL** | **TRIG** | **Ca** | **Cl** | **K** | **Na** | **GLOB** | **AG** |  |  |
| **Vehicle** | Mean | 29 | 83 | 197 | 1.37 | 54 | 29 | 32 | 4.6 | 8.57 | 1.52 | 0.54 | 2.63 | 101.6 | 3.94 | 140 | 24 | 1.22 |  |  |
|  | SEM | 4 | 7 | 25 | 0.11 | 1 | 1 | 3 | 0.6 | 0.68 | 0.1 | 0.12 | 0.03 | 1.2 | 0.19 | 1 | 1 | 0.04 |  |  |
|  | N | 4 | 5 | 5 | 5 | 5 | 5 | 5 | 5 | 5 | 5 | 5 | 5 | 5 | 5 | 5 | 5 | 5 |  |  |
| **BRT_002 (100 mg/kg/day)** | Mean | 28 | 69 | 172 | 1.41 | 55 | 29 | 32 | 4.3 | 10.25 | 1.18 | 1.07 | 2.64 | 100.8 | 4.14 | 139 | 26 | 1.11 |  |  |
|  | SEM | 1 | 4 | 27 | 0.09 | 1 | 0 | 2 | 0.3 | 0.46 | 0.04 | 0.3 | 0.06 | 0.8 | 0.29 | 1 | 1 | 0.05 |  |  |
|  | % | 4 | 4 | 4 | 4 | 5 | 5 | 5 | 5 | 5 | 5 | 5 | 5 | 5 | 5 | 5 | 5 | 5 |  |  |
|  | N | -3 | -17 | -13 | 3 | 2 | 0 | 0 | -7 | 20 | -22 | 98 | 0 | -1 | 5 | -1 | 8 | -9 |  |  |
|  | P | NS | NS | NS | NS | NS | NS | NS | NS | NS | * | NS | NS | NS | NS | NS | NS | NS |  |  |
|  | Threshold | 11 | 19 | 103 | 0.3 | 4 | 1 | 8 | 1.4 | 2.24 | 0.32 | 0.7 | 0.16 | 3.5 | 0.93 | 3 | 4 | 0.15 |  |  |

**Supplementary Table 2:** **Bioaccumulation study.** BLD: below the limit of detection; BLLQ: below the lower limit of quantification; lower limit of quantification: 0.924 ng/g; upper limit of quantification: 1849 ng/g.

|  | **Concentration of BRT_001 (ng/g of tissue)** | | | | | | | |
| --- | --- | --- | --- | --- | --- | --- | --- | --- |
| **Treatment** | **Animal ID** | **Brain** | **Liver** | **Heart** | **Spleen** | **Kidney** | **Lung** | **Spinal cord** |
| **Vehicle** | 1 | BLD | BLD | BLD | BLD | BLD | BLD | BLD |
|  | 2 | BLD | BLD | BLD | BLD | BLD | BLD | BLD |
| **BRT_002 (100 mg/kg/day)** | 3 | 0.966 | 178 | 21.9 | 17.3 | 733 | 1517 | 22.2 |
|  | 4 | BLD | 70 | 107 | 12.1 | 159 | 481 | 9.48 |

**Supplementary Table 3:** **Pharmacokinetic (PK) parameters of BRT_001.** Cmax: Maximal Concentration; Tmax: Time to obtain maximal concentration; AUC: Area Under the Curve; t_1/2_: Half-life of elimination; Vz: volume of distribution; Vss: Volume of distribution at steady-state; F: absorbed fraction; Clast (Obs): Last concentration observed; TLast: Time of last concentration observed.

|  |  | **PK Parameters** | **Cmax** | **Tmax** | **AUC 0-24h** | **AUCtot** | **%AUCextra** | **t_1/2_** | **Clearance** | **Vz** | **Vss** | **CLast(Obs)** | **TLast** |
| --- | --- | --- | --- | --- | --- | --- | --- | --- | --- | --- | --- | --- | --- |
|  |  | **Unit** | ng/mL | h | ng/mL*h | ng/mL*h |  | h | mL/min/kg | L/kg | L/kg | ng/mL | h |
| **Serum** | | Sham male | 635.5 | 1 | 3596.69 | 3626.59 | 0.82455 | 3.42071 | 101.702 | 30.1143 | 35.3028 | 5.811 | 24 |
|  |  | Sham female | 700.7 | 1 | 2565.93 | 2572.67 | 0.261921 | 2.768 | 143.366 | 34.3509 | 37.9636 | 1.67 | 24 |
|  |  | HI male | 816.1 | 1 | 2399.75 | 2451.34 | 2.1049 | 4.40001 | 150.462 | 57.3067 | 48.6672 | 8.697 | 24 |
|  |  | HI female | 575.5 | 2 | 2959.65 | 3009.92 | 1.66986 | 3.92489 | 122.539 | 41.6322 | 42.5632 | 9.46 | 24 |
| **Brain** | **Contralateral hemisphere** | Sham male | 609.2 | 1 | 2528.24 | 2534.47 | 0.245827 | 2.76698 | 197.28 | 47.2514 | 59.9878 | 1.418 | 24 |
|  |  | Sham female | 613,0 | 1 | 1762.61 | 1764.93 | 0.131716 | 2.51407 | 283.297 | 61.6517 | 69.8918 | 0.6089 | 24 |
|  |  | HI male | 926.8 | 1 | 2521.98 | 2545.8 | 0.935821 | 3.6522 | 196.402 | 62.0906 | 56.4011 | 4.582 | 24 |
|  |  | HI female | 637.2 | 1 | 3055.07 | 3083.22 | 0.91289 | 3.44981 | 162.168 | 48.4269 | 53.246 | 5.73 | 24 |
|  | **Ipsilateral hemisphere** | Sham male | 667.6 | 1 | 2292.17 | 2301.29 | 0.396226 | 3.10155 | 217.269 | 58.3315 | 70.3897 | 1.799 | 24 |
|  |  | Sham female | 591.3 | 1 | 1816.92 | 1817.28 | 0.0197888 | 1.90577 | 275.136 | 45.3883 | 59.486 | 0.1243 | 24 |
|  |  | HI male | 553 | 1 | 2330.49 | 2373.26 | 1.80202 | 4.13152 | 210.681 | 75.3462 | 75.557 | 7.256 | 24 |
|  |  | HI female | 463.9 | 1 | 2854.16 | 2913.46 | 2.03535 | 4.16382 | 171.617 | 61.8556 | 66.7138 | 9.99 | 24 |

**Supplementary Table 4: Distribution of the numbers of biological replicates in the sham, HI+Veh and HI+BRT_002 groups.**

| # | **Group** | **Label-status** | **Gender** | **Brain Hemisphere** | **Number of replicates** |
| --- | --- | --- | --- | --- | --- |
| 1 | Control  (Not Treated) | Sham | Female | Contralateral | 6 |
|  |  |  |  | Ipsilateral | 6 |
|  |  |  | Male | Contralateral | 3 |
|  |  |  |  | Ipsilateral | 3 |
| 2 | Control  (Placebo-Treated) | Hypoxia ischemia + Veh | Female | Contralateral | 6 |
|  |  |  |  | Ipsilateral | 6 |
|  |  |  | Male | Contralateral | 6 |
|  |  |  |  | Ipsilateral | 6 |
| 3 | BRT_002-Treated | Hypoxia ischemia + BRT_002 | Female | Contralateral | 5 |
|  |  |  |  | Ipsilateral | 5 |
|  |  |  | Male | Contralateral | 4 |
|  |  |  |  | Ipsilateral | 4 |

**Supplementary Table 5:** **Summary of differentially expressed proteins (DEPs) among neonatal rats in the sham, HI+Veh and HI+BRT_002 groups.**

| **#** | **Comparison** | **Sex** | **Brain Hemisphere** | **No. of DEPs**  **(p < 0.05)** | **Common DEPs** | |
| --- | --- | --- | --- | --- | --- | --- |
|  |  |  |  |  | **Upregulated** | **Downregulated** |
| 1 | HI+Veh vs. sham | F (6) vs. F (6) | Ipsilateral | 491 | 260 | 231 |
| 2 | HI+Veh vs. sham | F (6) vs. F (6) | Contralateral | 986 | 490 | 496 |
| 3 | HI+Veh vs. sham | M (6) vs. M (3) | Ipsilateral | 362 | 259 | 103 |
| 4 | HI+Veh vs. sham | M (6) vs. M (3) | Contralateral | 226 | 100 | 126 |
| 5 | HI+BRT_002 vs. HI+Veh | F (5) vs. F (6) | Contralateral | 399 | 200 | 199 |
| 6 | HI+BRT_002 vs. HI+Veh | F (5) vs. F (6) | Ipsilateral | 276 | 160 | 116 |
| 7 | HI+BRT_002 vs. HI+Veh | M (4) vs. M (6) | Contralateral | 320 | 130 | 190 |
| 8 | HI+BRT_002 vs. HI+Veh | M (4) vs. M (6) | Ipsilateral | 91 | 48 | 43 |
| 9 | HI-BRT_002 vs. HI-Veh | M (4) vs. F (6) | Contralateral | 341 | 12 | 23 |
|  | HI-BRT_002 vs. HI-Veh | M (6) vs. F (5) | Contralateral | 463 |  |  |
| 10 | HI-BRT_002 vs. HI-Veh | M (4) vs. F (6) | Ipsilateral | 83 | 3 | 7 |
|  | HI-BRT_002 vs. HI-Veh | M (6) vs. F (5) | Ipsilateral | 366 |  |  |

M: male; F: female. In the column for sex, the number of biological replicates in the experiment is presented in parentheses.

**Supplementary Table 6: List of dysregulated proteins common to the ipsilateral and contralateral brain hemispheres of BRT_002-treated female neonatal rats with moderate HI.**

| **#** | **Protein ID** | **Protein name** |
| --- | --- | --- |
| 1 | Ap2b1 | Adaptor related protein complex 2 subunit beta 1 |
| 2 | Ap2m1 | Adaptor related protein complex 2 subunit mu 1 |
| 3 | Bzw1 | Basic leucine zipper and W2 domains 1 |
| 4 | Copb1 | COPI coat complex subunit beta 1 |
| 5 | Copb2 | COPI coat complex subunit beta 2 |
| 6 | Copg1 | COPI coat complex subunit gamma 1 |
| 7 | Coro7 | Coronin 7 |
| 8 | Dpp8 | Dipeptidylpeptidase 8 |
| 9 | Dynll2 | Dynein light chain LC8-type 2 |
| 10 | Eef2 | Eukaryotic translation elongation factor 2 |
| 11 | Eif3b | Eukaryotic translation initiation factor 3, subunit B |
| 12 | Ide | Insulin degrading enzyme |
| 13 | Ipo5 | Importin 5 |
| 14 | Ipo7 | Importin 7 |
| 15 | Lonp1 | lon peptidase 1, mitochondrial |
| 16 | Macf1 | Microtubule-actin crosslinking factor 1 |
| 17 | Mms19 | MMS19 homolog, cytosolic iron-sulfur assembly component |
| 18 | Pfdn2 | Prefoldin subunit 2 |
| 19 | Psmd1 | Proteasome 26S subunit, non-ATPase 1 |
| 20 | Psmd2 | Proteasome 26S subunit ubiquitin receptor, non-ATPase 2 |
| 21 | Rab12 | RAB12, member RAS oncogene family |
| 22 | Rpl26 | Ribosomal protein L26 |
| 23 | Sephs1 | Selenophosphate synthetase 1 |
| 24 | Sf3b3 | Splicing factor 3b, subunit 3 |
| 25 | Snd1 | Staphylococcal nuclease and tudor domain containing 1 |
| 26 | Snrpa | Small nuclear ribonucleoprotein polypeptide A |
| 27 | Strip1 | Striatin interacting protein 1 |
| 28 | Thop1 | Thimet oligopeptidase 1 |
| 29 | Uba1 | Ubiquitin-like modifier activating enzyme 1 |
| 30 | Ube2i | Ubiquitin-conjugating enzyme E2I |
| 31 | Ube4a | Ubiquitination factor E4A |
| 32 | Usp5 | Ubiquitin specific peptidase 5 |
| 33 | Vapa | VAMP associated protein A |
| 34 | Xpo7 | Exportin 7 |

**Supplementary Table 7: List of dysregulated proteins common to the ipsilateral and contralateral brain hemispheres of BRT_002-treated male neonatal rats with moderate HI.**

| # | **Protein ID** | **Protein name** |
| --- | --- | --- |
| 1 | Atp5if1 | ATP synthase inhibitory factor subunit 1 |
| 2 | Bsg | Basigin (Ok blood group) |
| 3 | Cadm2 | Cell adhesion molecule 2 |
| 4 | Coro1b | Coronin 1B |
| 5 | Cyp51 | Cytochrome P450, family 51 |
| 6 | Dynlt1 | Dynein light chain Tctex-type 1 |
| 7 | Eml2 | EMAP like 2 |
| 8 | Epb41l2 | Erythrocyte membrane protein band 4.1-like 2 |
| 9 | Fv1 | Friend virus susceptibility 1 |
| 10 | Pals2 | Protein associated with LIN7 2, MAGUK p55 family member |
| 11 | Septin8 | Septin 8 |
| 12 | Sms | Spermine synthase |

**Supplementary Table 8: qPCR primers.**

| **Gene name** | **Forward 5’-3’** | **Reverse 5’-3’** |
| --- | --- | --- |
| ***Gfap*** | GCTAATGACTATCGCCGCCAAC | GCATTTGCCTCTCCAAGGACTC |
| ***Iba1*** | ATCGTCATCTCCCCACCTAAGG | TCCCATCCAACCTCTCTTCCTG |
| ***Map2*** | CGGAAAACCACAGCAACAAGT | GGTCTTGGGAGGGAAGAACG |
| ***NeuN*** | AGCACAGACAGATAGCCAGC | TTTCCCGAATTGCCCGAACA |
| ***Vegfa*** | CCACTTCTGAGGAGCCTAG | GGAGGAGGAGCCATTACC |
| ***Angt1*** | TGCTAACAGGAGGTTGGTGG | TTTTCCATGGTTTTGCCCCG |
| ***Angt2*** | GACGGCTGTGATGATCGAGA | CGTCTGGTTTAGTACTTGGGCT |
| ***Apcdd1*** | CTGCAGAACGCCAAGAATCAC | CATTCCCCATGGAGGCCAAT |
| ***Pacs1*** | CCTGCAGGCATAAGTTCCCT | GGAGGGCGAGTCTTCAACAA |
| ***Pacs2*** | TCCTTTTGTCGGGGTTGTGAA | ATGTAGATGCAGACGGTGGG |
| ***Letm1*** | GGTTGACGAGCCATGAGAGT | CAGGCCTCTGAGTCCAACAG |
| ***Vwf*** | CCAGCCACATTCCATACAATC | CCAATCAACACAGACTCCATTAG |
| ***Slc17a7*** | ACAGCCTTTTGCGGTTCCTA | CCCGAAGCTGCCATAGACAT |
| ***Gabbr1*** | CTACAATGTCGCGGTCCTGT | GGCACAAAGAGCACAACCAG |
| ***Txn2*** | ATTTGCCTCTGGTGTATTTC | TTATCCATTACTCTCACTTGTTC |
